# Auxiliary α2δ1 and α2δ3 Subunits of Calcium Channels Drive Excitatory and Inhibitory Neuronal Network Development

**DOI:** 10.1523/JNEUROSCI.1707-19.2020

**Published:** 2020-06-17

**Authors:** Arthur Bikbaev, Anna Ciuraszkiewicz-Wojciech, Jennifer Heck, Oliver Klatt, Romy Freund, Jessica Mitlöhner, Sara Enrile Lacalle, Miao Sun, Daniele Repetto, Renato Frischknecht, Cornelia Ablinger, Astrid Rohlmann, Markus Missler, Gerald J. Obermair, Valentina Di Biase, Martin Heine

**Affiliations:** ^1^RG Functional Neurobiology, Institute for Developmental Biology and Neurobiology, Johannes Gutenberg University Mainz, Mainz, 55128, Germany; ^2^RG Molecular Physiology, Leibniz Institute for Neurobiology, Magdeburg, 39118, Germany; ^3^Center for Behavioral Brain Sciences, Otto-von-Guericke University Magdeburg, Magdeburg, 39106, Germany; ^4^Institute for Anatomy and Molecular Neurobiology, University of Münster, Münster, 48149, Germany; ^5^RG Brain Extracellular Matrix, Leibniz Institute for Neurobiology, Magdeburg, 39118, Germany; ^6^Department of Biology, Animal Physiology, Friedrich Alexander University of Erlangen-Nuremberg, Erlangen, 91058, Germany; ^7^Institute of Physiology, Medical University Innsbruck, Innsbruck, 6020, Austria; ^8^Division Physiology, Karl Landsteiner University of Health Sciences, Krems, 3500, Austria; ^9^Institute of Molecular and Clinical Pharmacology, Medical University Innsbruck, Innsbruck, 6020, Austria

**Keywords:** alpha2delta subunits, excitation to inhibition balance, network connectivity, synaptogenesis, VGCCs

## Abstract

VGCCs are multisubunit complexes that play a crucial role in neuronal signaling. Auxiliary α2δ subunits of VGCCs modulate trafficking and biophysical properties of the pore-forming α1 subunit and trigger excitatory synaptogenesis. Alterations in the expression level of α2δ subunits were implicated in several syndromes and diseases, including chronic neuropathic pain, autism, and epilepsy. However, the contribution of distinct α2δ subunits to excitatory/inhibitory imbalance and aberrant network connectivity characteristic for these pathologic conditions remains unclear. Here, we show that α2δ1 overexpression enhances spontaneous neuronal network activity in developing and mature cultures of hippocampal neurons. In contrast, overexpression, but not downregulation, of α2δ3 enhances neuronal firing in immature cultures, whereas later in development it suppresses neuronal activity. We found that α2δ1 overexpression increases excitatory synaptic density and selectively enhances presynaptic glutamate release, which is impaired on α2δ1 knockdown. Overexpression of α2δ3 increases the excitatory synaptic density as well but also facilitates spontaneous GABA release and triggers an increase in the density of inhibitory synapses, which is accompanied by enhanced axonaloutgrowth in immature interneurons. Together, our findings demonstrate that α2δ1 and α2δ3 subunits play distinct but complementary roles in driving formation of structural and functional network connectivity during early development. An alteration in α2δ surface expression during critical developmental windows can therefore play a causal role and have a profound impact on the excitatory-to-inhibitory balance and network connectivity.

**SIGNIFICANCE STATEMENT** The computational capacity of neuronal networks is determined by their connectivity. Chemical synapses are the main interface for transfer of information between individual neurons. The initial formation of network connectivity requires spontaneous electrical activity and the calcium channel-mediated signaling. We found that, in early development, auxiliary α2δ3 subunits of calcium channels foster presynaptic release of GABA, trigger formation of inhibitory synapses, and promote axonal outgrowth in inhibitory interneurons. In contrast, later in development, α2δ1 subunits promote the glutamatergic neurotransmission and synaptogenesis, as well as strongly enhance neuronal network activity. We propose that formation of connectivity in neuronal networks is associated with a concerted interplay of α2δ1 and α2δ3 subunits of calcium channels.

## Introduction

The transfer and processing of information in neuronal networks critically depend on structural and functional connections between neurons. Network connectivity is not static but evolves over time and reflects both genetically predetermined factors and the previously processed stimuli. The initial circuitry formation occurs during early development and is associated with the emergence of synaptic contacts, which serve as substrate for functional network interaction. During early development, spontaneous neuronal activity involving transient changes in intracellular calcium is necessary and sufficient for neuronal development, and powerfully drives the establishment of connectivity maps (Ben-Ari, 2001; Spitzer, 2006).

VGCCs (Ca_V_s) on presynaptic boutons play a crucial role in synaptic transmission by mediating the electrochemical conversion of electrical activity into vesicle release. VGCCs are multiunit complexes that consist of a mandatory pore-forming α1 subunit and auxiliary α2δ and β subunits (Catterall, 2000; Arikkath and Campbell, 2003; Zamponi et al., 2015). In mammalian synapses, activation of mainly P/Q-type (Ca_V_2.1) and N-type (Ca_V_2.2) VGCCs on membrane depolarization results in rapid presynaptic calcium influx that triggers neurotransmitter release (Wheeler et al., 1994; Scholz and Miller, 1995; Cao and Tsien, 2010). Four α2δ isoforms (α2δ1-4) encoded by *CACNA2D1-CACNA2D4* genes have been identified, with α2δ1 and α2δ3 being particularly abundant in the cerebral cortex and hippocampus (Klugbauer et al., 1999; Cole et al., 2005; Schlick et al., 2010). Expression of the β and α2δ subunits increases the trafficking of the channel and modulates its biophysical properties at the surface (Arikkath and Campbell, 2003; Dolphin, 2012). For example, overexpression of α2δ subunits triggers synaptic recruitment of VGCCs, enlargement of the presynaptic terminals, and facilitation of presynaptic release (Hoppa et al., 2012; Schneider et al., 2015), whereas downregulation of α2δ subunits decreases the surface expression of α1 subunit and leads to the reduction of presynaptic structures and glutamate release (Dickman et al., 2008; Kurshan et al., 2009; Cordeira et al., 2014). Additionally, α2δ1 and α2δ3 subunits were shown to promote excitatory synaptogenesis in mammalian brain (Eroglu et al., 2009) and in *Drosophila* (Dickman et al., 2008; Kurshan et al., 2009), respectively.

Altered expression of α2δ subunits has been implicated in the pathogenesis of several syndromes and diseases (Geisler et al., 2015; Zamponi et al., 2015). In particular, postinjury overexpression of α2δ1 in sensory neurons is associated with hyperalgesia and chronic neuropathic pain and underlies the antiallodynic efficacy of gabapentinoids (Luo et al., 2001; Bauer et al., 2009; Patel et al., 2013). Null mutation of *CACNA2D2* leads to global developmental delay, absence epilepsy, and cerebellar ataxia in mice (Barclay et al., 2001) and humans (Edvardson et al., 2013; Pippucci et al., 2013). Symptomatic convulsive epilepsy and intellectual disability were also reported in humans with aberration of the *CACNA2D1* gene (Vergult et al., 2015). Furthermore, analyses of gene-disrupting mutations in individuals with autism highlighted *CACNA2D3* among autism susceptibility genes (Iossifov et al., 2012; De Rubeis et al., 2014). Autism is a pervasive neurodevelopmental disorder diagnosed early in childhood and associated with aberrant brain connectivity (Folstein and Rosen-Sheidley, 2001; Freitag, 2007). Remarkably, autistic spectrum disorders are accompanied by epilepsy in up to 38% of affected individuals, which represents manifold higher incidence of epilepsy compared with the population average (Tuchman and Rapin, 2002; Levisohn, 2007).

Thus, converging lines of evidence suggest that α2δ subunits are involved in the establishment and/or modulation of the excitation/inhibition ratio, but little is known about the mechanisms and the contribution of individual α2δ isoforms to network connectivity and activity of central neurons. Therefore, in this study, we used acute upregulation and downregulation of the α2δ subunits to dissect their impact on the formation of structural and functional connectivity, as well as on the balance between excitation and inhibition.

## Materials and Methods

### 

#### Ethics statement

All experimental procedures were conducted in accordance with the EU Council Directive 86/609/EEC and were approved and authorized by the local Committee for Ethics and Animal Research (Landesverwaltungsamt Halle, Germany).

#### Breeding and genotyping of mutant mice

Animal procedures for control and α2δ1 KO (α_2_δ1^−/−^) mice having a mixed 129J × C57BL/6 background were performed at the Medical University Innsbruck in compliance with government regulations and approved by the Austrian Federal Ministry of Science, Research and Economy (license #BMWFW-66.011/0113-WF/V/3b/2014 and #BMWFW-66.011/0114-WF/V/3b/2014). Regular reports including the mouse numbers used for thisproject were given to the Austrian Federal Ministry of Science, Research and Economy (BMWFW). Animal experiments at the University of Münster involving WT mice were performed in accordance with government regulations for animal welfare and approved by the Landesamt für Natur, Umwelt und Verbraucherschutz (license #84-02.05.20.11.209 and #84-02.04.2015.A423). Mice were maintained at central animal facilities in Innsbruck and Münster under standard housing conditions with food and water *ad libitum* at a 12 h light/dark cycle. The α2δ1^−/−^ mutant mouse strain was previously generated and characterized (Fuller-Bicer et al., 2009; Patel et al., 2013; Mastrolia et al., 2017). Genotyping for the Cacna2d1 gene was done as published previously (Fuller-Bicer et al., 2009) with some modifications by use of standard PCR conditions (annealing at 52°C for 30 s). Primers: WT-F1: 5′-GAGCTTTCTTTCTTCTGATTCCAC-3′, mutant-F2: 5′-CTGCACGAGACTAGTGAGACG-3′, R: 5′-ACATTCTCAAGACTGTAGGCAGAG-3′. Expected band sizes were 346 bp for WT (α2δ1^+/+^) and 635 bp for KO (α2δ1^−/−^) animals, respectively, and heterozygous mice showed both bands.

#### Transmission electron microscopy

Brain tissue from WT control and α2δ1^−/−^ mice was embedded in epon resin (Electron Microscopy Science). For embedding, anesthetized adult male mice were transcardially perfused with 25 ml of 2% glutaraldehyde (Roth) and 2% PFA (Merck) in 0.1 M PB at 37°C, and postfixed at 4°C overnight. Blocks of hippocampal tissue were contrasted in 1% osmium tetroxide for 2 h at room temperature. Following washes with distilled water and dehydrating, tissue was incubated with propylene oxide (Electron Microscopy Science) for 45 min, infiltrated with propylene oxide/epon (1:1) for 1 h, in pure epon overnight, and hardened at 60°C for 24 h. Additional contrasting of thin sections from brains was done on Formvar-coated copper grids with a saturated solution of 12% uranyl acetate and lead citrate.

For better comparability with imaging and electrophysiological results, samples containing the stratum radiatum of the hippocampal CA1 region were investigated. Ultrastructural analysis was done with a transmission electron microscope (Libra 120, Carl Zeiss) at 80 kV, and images taken with a CCD camera (Tröndle). For quantifying the density of asymmetric synapses, tissue areas were reconstructed from panorama pictures (each composed of 9 individual images = 210 µm^2^), and three panoramas were analyzed per genotype (*n* = 3 panoramas from 3 animals per genotype = 1890 µm^2^). Asymmetric (Type 1) synapses were defined as contacts with a visible synaptic cleft, a distinct postsynaptic density, and at least three synaptic vesicles.

##### Cloning of lentiviral α2δ::HA overexpression constructs

For immunoreactive detection, α2δ subunits were N-terminally labeled with a double hemagglutinin (HA)-tag. The extracellularly double HA-labeled (between aa 27 and 28) rabbit α2δ1 construct was kindly provided by G.J.O. (Medical University Innsbruck). For the α2δ3, the double HA-tag was inserted between aa 36 and 37 of mouse CACNA2D3 (provided by Prof. Norbert Klugbauer, Albert-Ludwigs-University Freiburg) (see Klugbauer et al., 1999) via a synthesized DNA fragment using the KpnI and BsrGI restriction sites. For cloning of lentiviral transfer plasmids for α2δ overexpression, a pLenti vector of the third generation equipped with a neuron-specific synapsin promotor was used as backbone (pLenti-Synapsin-hChR2(H134R)-EYFP-WPRE; Addgene; plasmid #20945). The hChR2 insert was cut from this vector via the unique sites AgeI and BsrGI, and sticky ends were used for insert integration or filled up to blunt ends using Klenow Fragment (Thermo Fisher Scientific). The α2δ1-2HA was enzymatically digested via the unique restriction sites NotI and SalI, filled up to blunt ends, and ligated into the lentiviral transfer vector. The α2δ3-2HA was amplified via PCR and equipped with the unique restriction sites BsiWI (generating BsrGI overhang) and AgeI allowing sticky-end ligation into the target vector. Correct integration was determined by qualitative digestion and partial sequencing.

##### Constructs for shRNA-mediated α2δ knockdown

For knockdown of the α2δ1 subunit, siRNA target sequences corresponding to the α2δ1 coding region (CACNA2D1, GenBank accession number NM_009784.2) (Obermair et al., 2005) were selected and tested for efficient knockdown. The siRNA was expressed as shRNA under the control of a U6 promoter (derived from the pSilencer1.0-U6 siRNA expression vector, Ambion) cloned into the pβA-eGFP plasmid (Obermair et al., 2010). For lentiviral expression, α2δ1 shRNA was cloned into pHR as previously described (Subramanyam et al., 2009). For knockdown of the α2δ3 subunit, four 29mer shRNA constructs against rat Cacna2d3 (Gene ID 306243) cloned in lentiviral GFP vector (pGFP-C-shLenti Vector, catalog #TR30023) were ordered from OriGene Technologies (catalog #TL713428). Based on their specificity for rat and mouse α2δ3, two of these constructs were tested for their knockdown efficiency where the construct “C” was evaluated to results in a reduction of α2δ3 expression down to 40%-50%. As control for α2δ-knockdown experiments, a noneffective 29-mer scrambled shRNA cassette cloned into pGFP-C-shLenti vector (catalog #TR30021; OriGene Technologies) was used.

##### Evaluation of shRNA-mediated α2δ3 knockdown

The knockdown efficiency of the α2δ3 shRNA constructs was tested on both the expression of HA-tagged α2δ3 (transfected into HEK293T cells and rat hippocampal cultures) and the endogenous expression level of α2δ3 in rat hippocampal cultures. Expression levels of HA-tagged α2δ3 subunits were quantified via anti-HA immunostaining and Western blotting (rat anti-HA, 1:1000, Roche, catalog #11867423001, clone 3F10) or monoclonal mouse anti-HA-tag (1:1000; OriGene Technologies, catalog #TA180128) and polyclonal anti-HA-tag (1:1000; Synaptic Systems, catalog #245003), respectively. Furthermore, HA-tagged α2δ3 subunits were used to evaluate the correct endogenous α2δ3 bands targeted in Western blotting via polyclonal rabbit anti CACNA2D3 (1:1000; Thermo Fisher Scientific, catalog #PA5-87 802). Protocols used for immunocytochemical staining and Western blotting are described below.

##### Preparation of cell lysates

For the validation of a2δ antibodies, HEK293T cells were transfected with HA-tagged a2δ1 and a2δ3 variants. Transfected cells as well as nontransfected HEK293T cells were processed for Western blot analysis 48 h after transfection. Cells were washed with ice-cold 1 × PBS for 2 times, scraped off, collected, and centrifuged at 800 rpm for 10 min. Afterward, cells were lysed with lysis buffer (125 mm sodium chloride, 0.1% [w/v] SDS, 0.01% [v/v] Triton X-100, 50 mm Tris/HCl, pH 7.5) containing a protease inhibitor cocktail (cOmplete ULTRA Tablets, Sigma Millipore, catalog #05892791001, Roche). Lysates were cleared by centrifugation at 15,000 rpm for 15 min at 4°C and incubated for 10 min at room temperature with 4 × loading buffer (40% [v/v] glycerol, 240 mm Tris/HCl, pH 6.8, 8% [w/v] SDS, 0.04% [w/v] bromophenol blue, 5% [v/v] β-mercaptoethanol). Primary neurons were infected with the overexpression or knockdown constructs 7 d before harvesting. In general, cells were harvested at DIV21-DIV28, except the a2δ3 knockdown condition was harvested at DIV11-DIV12 where the a2δ3 expression was found to be most prominent. For sample collection, cells were washed with prewarmed 1× PBS and directly lysed using 2× sample buffer (1× Tris/HCl, pH 6; 8. 4× Tris/HCl, 500 mm Tris, 0.4% [w/v] SDS), 20% [v/v] glycerol, 4% [w/v] SDS, 2% [v/v] β-mercaptoethanol and 0.001% [w/v] bromophenol blue) containing a protease inhibitor cocktail (cOmplete ULTRA Tablets, Sigma Millipore, catalog #05892791001, Roche). Cells were then scraped and the lysate was pipetted up and down (at least 5 times) through a 30 G cannula. The lysate was then incubated for 1 h at 37°C and briefly spun down before gel loading.

##### Western blotting

Samples were loaded on a 5% acrylamide stacking gel and separated by 1D SDS-PAGE under fully denaturing conditions. Tris-glycine gels (containing trichloroethanol) were prepared with a gradient of 5% acrylamide (at the top) and 20% (at the bottom). Afterwards, gels were activated using UV light to provoke an excited-state reaction of tryptophan amino acids of the separated proteins with trichloroethanol-producing fluorescence in the visible range. The electrophoretic transfer onto a PVDF membrane (Carl Roth, catalog #T830.1) was performed according to standard protocols, and the transferred total protein fraction was acquired with UV light. Membranes were briefly washed with 1× TBS-T and subsequently blocked with 5% [w/v] milk (Carl Roth, catalog #T145.2) in 1 × TBS-T (50 mm Tris/HCl, 150 mm NaCl, 0.1% [v/v] Tween-20, pH 7.5) for 30 min at room temperature. Primary antibodies, targeting the respective HA-tagged or endogenous a2δ protein of interest as well as the loading control β-actin, were diluted (as indicated) in 5% [w/v] milk and incubated overnight at 4°C: monoclonal mouse anti-HA-tag (1:1000; OriGene Technologies, catalog #TA180128), polyclonal anti-HA-tag (1:1000; Synaptic Systems, catalog #245003), polyclonal rabbit anti human Cacna2d1 (1:200; Alomone Labs, catalog #ACC-015), polyclonal rabbit anti-Cava2δ3 (extracellular) (1:200; Santa Cruz Biotechnology, catalog #sc-99 324), polyclonal rabbit anti-CACNA2D3 (1:1000; Thermo Fisher Scientific, catalog #PA5-87 802), and monoclonal mouse anti-β-actin (1:2000; Synaptic Systems, catalog #251011). Afterward, membranes were washed 3 times with 1× TBS-T and incubated with secondary antibodies coupled to NIR fluorophores (AlexaFluor-680 goat anti-rabbit, 1:10,000; Thermo Fisher Scientific, catalog #A27042; and AlexaFluor-790 donkey anti-mouse, 1:10,000; Dianova, catalog #715-655-150) or coupled to HRP (peroxidase-conjugated AffiniPure goat anti-mouse IgG [H + L]; 1:1000; Jackson ImmunoResearch Laboratories, catalog #115-035-146; or peroxidase-conjugated AffiniPure donkey anti-rabbit IgG [H + L]; 1:1000; Jackson ImmunoResearch Laboratories, catalog #711-035-152) for 45-60 min. Protein detection was performed using a LI-COR Odyssey scanner (for NIR) or Intas NEW-Line ECL ChemoStar Touch Imager HR 9.0 (for HRP). Protein quantification was performed with Fiji ImageJ 2.0.0-rc-69/1.52n.

For Western blots, quantification of presynaptic markers, P40-P60 brains from WT, and α2δ-1 KO mice were lysed in 50 mm Tris-HCl, pH 7.5, 80 mm NaCl, 1% Triton X-100, supplemented with 1 mm PMSF and protease inhibitor cOmplete (Roche). Briefly, brains were mashed in lysis buffer with Polytron (Kinematica AG) at 22,000 rpm until complete tissue dissociation and subsequently centrifuged at 700 × *g* for 5 min at 4°C. After 2 h lysis by rotation at 4°C, supernatants were collected and centrifuged at 220,000 × *g* for 30 min at 4°C. Supernatants were diluted in 2× loading buffer, and 20 μl was loaded on 8% and 10% acrylamide/bis-acrylamide gels. Proteins were transferred on PVDF membranes (Roth), blocked with TBS 0.3% Tween 5% BSA for 1 h at room temperature, and incubated overnight with the following antibodies: anti-Synapsin1a/1b (Synaptic Systems, catalog #106001) 1:500, anti-Rab3A (Sigma Millipore, catalog #R2776) 1:500, anti-synaptophysin (Synaptic Systems, catalog #101002) 1:500, anti-synaptotagmin1 (Synaptic Systems, catalog #105102) 1:500, anti-VGlut1 (Synaptic Systems, catalog #13551) 1:500, anti GAD65 (Abcam, catalog #Ab 85 866) 1:1000, anti-TH (Synaptic Systems, catalog #213111) 1:500, anti-SNAP-25 (Synaptic Systems, catalog #111001), anti- Cav2.1 P/Q type (Synaptic Systems, catalog #152203) 1:1000, anti-CASK (Abnova, catalog #PAB2776) 1:500, anti-liprinα3 (Synaptic Systems, catalog, #169102), anti-actin (Santa Cruz Biotechnology, catalog #SC-56 459) 1:500, and anti-vinculin (Santa Cruz Biotechnology, catalog #SC73614) 1:500.

##### Immunocytochemistry

The immunostaining was performed on HEK293T cells and rat hippocampal cultures grown on coverslips as described previously (Schneider et al., 2015). Briefly, cells were fixed in 4% PFA in 1× PBS for 5 min and subsequently permeabilized for 2 min with 0.3% Triton-X in 1× PBS. Afterward, cells were washed 3 times for 10 min with a buffer solution containing 25 mm glycine and 2% BSA in 1× PBS, and primary and secondary antibodies were applied consecutively for 1 h at room temperature. After additional washing steps, cells were mounted on glass slides with Mowiol (9.6 g; 24 ml H_2_O; 24 g glycerol; 48 ml 0.2 M Tris/HCl, pH 8.5). The following primary antibodies were used: rat anti-HA 1:1000 (Roche, catalog #11867423001, clone 3F10), mouse anti-HA at 1:1000 (Covance, catalog #MMS-101P, clone 16B12), guinea pig polyclonal anti-Bassoon at 1:1000 (Synaptic Systems, catalog #141004), rabbit polyclonal anti-Homer1 at 1:1000 (Synaptic Systems, catalog #160003), rabbit anti-gephyrin at 1:1000 (Synaptic Systems, catalog #147111), and secondary antibodies fluorescently labeled with Alexa-488, Alexa-568, Cy3, Alexa-647, or Cy5 (Jackson ImmunoResearch Laboratories, Thermo Fischer Scientific). The analysis of synaptic density and fluorescence intensity was performed using in ImageJ software (National Institutes of Health).

For image acquisition, *z* stacks were acquired for 20 planes at 200 nm steps, using a spinning disk confocal microscopy system (Andor Technology) controlled by Andor iQ2 software. The microscope (BX51WI Olympus) was equipped with a CSU-X1 spinning disk (Yokogawa), an EMCCD camera (iXon+ 897, Andor Technology), and 60 ×, NA 1.4 oil objective (Olympus) for synaptic density analysis or using a 20 ×, 0.8 NA oil objective (Olympus) to investigate axonal outgrowth.

##### Lentivirus production

For production of lentiviral particles, human embryonic kidney cells (HEK293T) cells were used for packaging and maintained in DMEM (Thermo Fisher Scientific) supplemented with 10% FCS (Thermo Fisher Scientific), 1% glutamine (Invitrogen), and 1 × antibiotic-antimycotic (Invitrogen) at 37°C in a humidified atmosphere with 5% CO_2_ and 95% air. The 30%-40% confluent HEK293T cells were triple-transfected with the second-generation helper plasmids: psPAX2 (Addgene, plasmid #12 260) and pVSV-G (Addgene, plasmid #8454), as well as the target gene-containing transfer vector in a molar ratio of 1:1:2. For the transfection of a 175 cm^2^ cell culture flask, 80 µg of total DNA was pipetted to 1 ml solution A (500 mm calcium chloride) and mixed. Subsequently, 1 ml of solution B (140 mm sodium chloride, 50 mm HEPES, 1.5 mm disodium hydrogen phosphate, pH 7.05) was added. The mixture was incubated for 2 min at room temperature and was then pipetted to the medium of the cells for overnight incubation. The next day, the medium was replaced by DMEM supplemented with only 4% FCS only, 1% glutamine, and 1 × antibiotic-antimycotic. On the following 2 d, the media was harvested, centrifuged at 2000 × *g* for 5 min, and the supernatant was stored at 4°C. Both harvests were pooled, filtered through a 0.45 µm filter, and centrifuged at 20,000 rpm for 2 h at 4°C. Afterward, the supernatant was removed and the pellet resuspended in DMEM (supplemented with 10% FCS, 1% glutamine, and 1 × antibiotic-antimycotic) on a shaker at 300 rpm and room temperature for 1 h.

##### Lentivirus titration

The working dilution of virus suspension was determined by test infection of dissociated EXVIII-EXIX rat cortical cultures seeded on coverslips and incubated in Neurobasal medium (Thermo Fisher Scientific) at 37°C in humidified atmosphere with 5% CO_2_ and 95% air. The cortical cultures were infected at DIV2 with dilutions of the viral particles from 1:50 to 1:5000, and incubated overnight. On the following day, the medium containing the virus was exchanged with the conditioned Neurobasal stored before infection. At DIV11, the cells were stained for α2δ expression via the HA-tag (rat anti-HA; Sigma Millipore, catalog #11867423001), cortical glial cells using anti-GFAP antibody (rabbit anti-GFAP; Synaptic Systems, catalog #173002), and total cell number via DAPI staining (0.5 mg/ml; Sigma Millipore, catalog #D9542). For this purpose, the cells were fixed for 5 min with 4% PFA (preheated to 37°C) and subsequently permeabilized with 0.3% Triton-X/PBS for 2 min at room temperature. Then, cells were washed three times for 10 min at room temperature using washing buffer (1× PBS; 2% BSA; 25 mm glycine) and incubated with the primary antibodies mentioned above at concentration of 1:1000 and DAPI 1:200 for 1 h at room temperature. Afterward, three washing steps were done, followed by the incubated with the secondary antibodies (1:1000): anti rat-Alexa-488 (Thermo Fisher Scientific, catalog #A11006) and anti-rabbit-Cy5 (Dianova, 111-175-144) for 1 h at room temperature in the dark. After three final washings, the coverslips were mounted with Mowiol (9.6 g Mowiol; 24 ml H_2_O; 24 g glycerol; 48 ml 0.2 M Tris/HCl, pH 8.5). Images were acquired with an Axio Imager.A2 microscope (Carl Zeiss) equipped with a CoolSNAP MYO CCD camera (Photometrics) and a 20× Plan-Apochromat oil objective (NA = 1.40, Carl Zeiss) using the VisiView (Visitron Systems) software. Images were acquired as stacks of 10 frames that were subsequently averaged and used for quantification in ImageJ (National Institutes of Health).

##### Heterologous expression of calcium channel subunits

Transient expression of tagged VGCCs in HEK293T was achieved by cotransfection of constructs for tagged α1 subunits together with β3- and α2δ1/3-encoding constructs at a 1:1:1 ratio using the FuGENE X-tremeGENE 9 DNA transfection reagent (Roche) according to the manufacturer's protocol. Transiently transfected cells were measured 48-72 h after transfection. Current amplitudes >1 nA were considered to result from successful cotransfection of all three subunits (α1, β3, and α2δ1/3) as confirmed further by simultaneous detection of the GFP-tag fused to β3 and the extracellular HA epitope in α2δ1/3::HA (data not shown).

##### Preparation, transfection, and infection of dissociated neuronal cultures

Dissociated hippocampal cultures were prepared from Wistar rat (Charles River; RRID:RGD_8553003) and glutamic acid decarboxylase 67 (GAD67)::GFP mouse embryos (EXVIII) as described previously (Kaech and Banker, 2006). Briefly, cell suspensions obtained after dissociation with trypsin were plated onto poly-L-lysine-coated 18 mm glass coverslips (Menzel-Glaeser) at a density of 30,000 cells per coverslip. After 1-2 h in DMEM plus FBS at 37°C, five coverslips were transferred into a 35 mm dish containing a 70%-80% confluent monolayer of astrocytes in Neurobasal medium supplemented with B27 and 5 mm glutamine. Cultures were incubated at 37°C in humidified atmosphere with 5% CO_2_ and 95% air. At DIV3, AraC was added to the cells to a final concentration of 1.4 μm.

For multichannel recordings, suspension of dissociated hippocampal cells (750,000 cells/ml) was plated on poly-D-lysine-coated 60-electrode microelectrode arrays (MEAs) with interelectrode distance 200 µm (MultiChannel Systems). After plating, all cultures were incubated at 37°C in humidified atmosphere (95% air and 5% CO_2_) in serum-free Neurobasal medium (Thermo Fisher Scientific). Throughout the lifespan, cultures were covered by semipermeable membranes (ALA-MEM, MultiChannel Systems) to avoid evaporation of the medium, which was partially replaced on a weekly basis.

Throughout the study, two infection protocols were applied. The first protocol was used to dissect the effects of α2δ subunits on the network activity during development and involved infection at distinct developmental stages (after first, second or third week *in vitro*) followed by recording of spontaneous activity 1 or 2 weeks later as specified in the text. The second protocol was used for analysis of long-term structural and functional consequences of upregulation or downregulation of α2δ subunits, as well as of lentiviral GFP expression. For this purpose, infection was performed during first week *in vitro* at DIV2-DIV4, and the data were acquired within the period of DIV7 to DIV24. Given the data on infection rate at different virus dilutions (data not shown), viral constructs were diluted in conditioned cultured media used for infections in the ratio 1:1000.

Transfection of neurons was conducted at DIV3-DIV4 using the calcium phosphate method. Before transfection, cells were placed in a 12-well dish with 1 ml 37°C Optimem media (Thermo Fisher Scientific). To prepare the precipitate, 150 μl of transfection buffer (in mM as follows: 274 NaCl, 10 KCl, 1.4 Na_2_HPO_4_, 15 glucose, 42 HEPES, pH 7.04-7.1) was added dropwise to a solution containing 5 μg of DNA and 200 mm CaCl_2_, under gentle stirring. The resulting mix was placed for 15 min at room temperature; 60 μl of the mix was added per well, and neurons were placed in the incubator for 30-60 min. Medium was exchanged for 2 ml 37°C prewarmed Neurobasal medium, followed by 2 times exchanging 1.5 ml. After this procedure, cells were finally placed back in the stored dishes in conditioned culture media.

##### Compounds and treatments

To test the contribution of Ca_V_2.2 and Ca_V_2.1 channels to mEPSCs and mIPSCs, specific calcium channel blockers ω-agatoxin IVA (200 nm) or ω-conotoxin GVIA (1 μm) (both from Alomone Labs), respectively, were applied to the bath solution. The changes of mPSCs frequency were analyzed ∼7 min after the toxin application. Contribution of high voltage-activated calcium channels to spontaneous neurotransmitter release was estimated by application of 100 μm CdCl_2_.

##### Synaptic density analysis

The analysis of synaptic density was conducted using custom-written routines for ImageJ software (National Institutes of Health). In rat hippocampal cultures infected with pLenti-syn-α2δ1::HA or pLenti-syn-α2δ3::HA, as well as in noninfected sister controls, immunolabeling of Bassoon and either Homer1 or Gephyrin was conducted for identification of presynaptic and postsynaptic sites of excitatory or inhibitory synapses, respectively. For each individual scan, puncta with the mean fluorescence exceeding arbitrary threshold value (2 SDs computed across the FOV) were detected and stored as sets of ROIs corresponding to individual presynaptic or postsynaptic compartments. Next, a segmented line was drawn by a trained user along individual dendrites from soma to the most distal point that could be reliably detected. Particularly in rather mature cultures with extensive dendritic branching, identification of individual dendrites was aided by additional MAP2 immunolabeling. The selected dendritic ROIs were straightened and the number of colocalized presynaptic and postsynaptic puncta per micrometer was calculated. For each preparation, the data obtained in α2δ-overexpressing cultures were further normalized to the mean value obtained in control sister cultures (taken as 100%).

##### Axonal outgrowth analysis

Axonal outgrowth was investigated either in WT rat hippocampal neurons transfected with the volume marker GFP or using hippocampal cultures of the GAD67-GFP mouse line (kindly provided by Prof. O. Stork, Otto-von-Guericke University of Magdeburg) to specifically examine GABAergic interneurons. In both cases, the GFP signal was enhanced via anti-GFP staining (Thermo Fisher Scientific, catalog #A6455). Neuronal dendrites were labeled using anti-MAP2 (Synaptic Systems, catalog #188004). The axons of GFP-positive neurons were identified based on the morphology of the neurites and negativity for MAP2 immunofluorescence.

In order to acquire the whole axons, 3-9 scans per neuron were taken. The analysis was done for each single image; values of images from the same cell were then integrated to obtain the total length and total number of branches per given axon. The measurement of axonal length and branching was performed by reconstructing the axons of acquired cells by using the Fiji plug-in Simple Neurite Tracer (Longair et al., 2011). The recording and analysis were conducted by a trained person in a blinded manner to exclude the bias in estimate of different conditions. For each preparation, the data obtained in α2δ-overexpressing cultures were further normalized to the mean value obtained in control sister cultures.

##### Whole-cell electrophysiological recordings

Recordings of recombinant VGCCs have been described previously (Brockhaus et al., 2018). In brief, patch-clamp recordings from transfected tsA-201 cells were done 3-5 d after plating. The bath solution (32°C) contained the following (in mm): 115 NaCl, 3 CaCl_2_, 1 MgCl_2_, 10 HEPES, glucose, 20 TEA-Cl, pH 7.4 (300 ± 5 mOsm/kg osmolality). Patch pipettes with a resistance of 2-4 MΩ when filled with pipette solution containing the following (in mm): 125 Cs-methane sulfonate, 20 TEA-Cl, 5 EGTA, 2 MgCl_2_, 10 HEPES, 4 Na_2_-ATP, 0.5 Na-GTP, pH 7.4 (285 ± 5 mOsm/kg osmolality). Whole-cell calcium currents were recorded with an EPC 10 USB Double patch-clamp amplifier and Patchmaster software (HEKA Elektronik). Signals were filtered at 3 kHz and digitized at 10 kHz. Cells were held at −80 mV in whole-cell configuration, series resistance, and membrane capacitance determined and compensated online. Leak currents were subtracted online using a P/5 protocol. Recordings for each condition were done on cells from at least three independent experiments. Current–voltage relationships were obtained by 50 ms voltage pulses from a holding potential of −80 mV to voltages between −40 mV and 70 mV in 10 mV increments with 6 s intervals. Current densities were calculated from currents normalized to whole-cell capacitance. Steady-state inactivation properties were measured by evoking currents with a 500 ms test pulse to 20 mV after 2 s voltage displacement (prepulse) from 20 mV to −80 mV in 10 mV increments (for further details, see Brockhaus et al., 2018).

The total whole-cell barium currents of high voltage-activated calcium channels from neuronal somata were recorded in extracellular solution with following composition (in mM):135 NaCl, 20 CsCl, 1 MgCl_2_, 5 BaCl_2_, 10 HEPES, 10 glucose, 5 4-diaminopyridine, and 0.0001 TTX (pH 7.3). Pipette solution contained in mM: 135 CsCl, 10 EGTA, and before experiments 1 ATP and 0.1 GTP were added to the pipette solution (pH 7.2). Barium currents were acquired from transfected hippocampal cultures (α2δ1::HA or α2δ3::HA) between DIV6 and DIV9.

Somatic whole-cell voltage-clamp recordings of spontaneous mEPSCs and mIPSCs were performed between DIV7 and DIV21 in cultured rat hippocampal neurons. Primary hippocampal cultures were constantly perfused with extracellular solution containing the following (in mM): 145 NaCl, 2.5 KCl, 2 MgCl_2_, 2 CaCl_2_, 10 HEPES, and 10 D-glucose (pH 7.4 adjusted with NaOH), supplemented with 0.1 μm TTX, 5 μm AP5, and 5 μm bicuculline (to record mEPSC) and 0.1 μm TTX, 5 μm AP5, and 10 μm DNQX (to record mIPSCs). Patch pipettes from borosilicate glass had a pipette resistance of 2-4 MΩ when filled with the intracellular solution of the following composition (in mM): 130 KCl, 2 MgCl_2_, 0.5 CaCl_2_, 1 EGTA, 40 HEPES (pH 7.25 adjusted with KOH). Before experiments, 1 mm ATP and 0.1 mm GTP were added, and pH was readjusted to 7.2-7.3 with KOH. Only patches with a series resistances <15 MΩ were analyzed. In all recordings, the membrane potential was clamped at −70 mV.

Individual mEPSCs and mIPSCs were detected using a peak detection algorithm of MiniAnalysis 6.0 software (Synaptosoft), which measured the peak amplitude, as well as rise and decay times. Amplitude threshold values were set at 3 times the root mean square of the baseline noise amplitude. All detected events were visually inspected and verified by a trained experimenter. The events were collected after 1-2 min after commencement of recording when the frequency of miniature currents was stabilized.

##### Recording and analysis of neuronal network activity

The neuronal network activity in high-density rat hippocampalcultures grown on MEAs was sampled at 10 kHz using MEA1060INV-BC system (MultiChannel Systems) at 37°C in a humidified atmosphere with 95% air and 5% CO_2_. The analysis was conducted using Spike2 software (Cambridge Electronic Design) on 10-min-long intervals for each culture at each time point. The threshold-based (±7 SDs of spike-free noise) detection of spikes in high pass-filtered records (gain 300 Hz) was followed by identification of bursts (≥5 spikes with interspike interval ≤ 100 ms). Channels with the mean firing rate lower than arbitrary minimum (0.01 spike/s) were considered as nonspiking in given session and discarded from further analyses. The mean firing rate was calculated separately for each active channel (electrode) in each individual culture. Network burst (NB) analysis was conducted as described previously (Bikbaev et al., 2015). NB was defined as a non-zero period of correlated (synchronous) bursting in two or more channels. For each NB, participating channels were ranked according to their temporal order of recruitment into given NB, forming vector (*1*, …, *n*), where *n* denotes the rank of the last recruited channel (i.e., the size of given NB; *n* ≥ 2). The mean burst onset lag reflecting the synchronicity of bursting onset in remote network locations was calculated for each NB as ${\Delta T}_{on}=\frac{\sum_{i=2}^{n}\left(T_{i}-T_{i-1}\right)}{\left(n-1\right)}$, where T*_i_* denotes the burst onset time in channel with rank *i* within given NB.

#### Statistics

To avoid potential bias of results, neuronal cultures grown on coverslips or MEAs were generally randomized before treatments. Additionally, experimental procedures and treatments, as well as separate experimental routines (acquisition, analysis, and interpretation) were conducted in a blind manner by different researchers where possible. The statistical effects of experimental treatments on analyzed parameters were evaluated using protected parametric and nonparametric (Kruskal-Wallis) ANOVA followed by *post hoc* tests as specified in the text. Pairwise comparisons were conducted using Student's *t* test or Mann–Whitney *U* test. Treatment of data and statistical analysis were performed using Prism software (GraphPad) and Statistica data analysis system (Statsoft). Factorial effects and differences were considered significant at *p* < 0.05. Data are presented as mean ± SEM.

## Results

### Constitutive KO of the α2δ1 subunit *in vivo* leads to reduction of excitatory synaptic density

**Figure 1.**
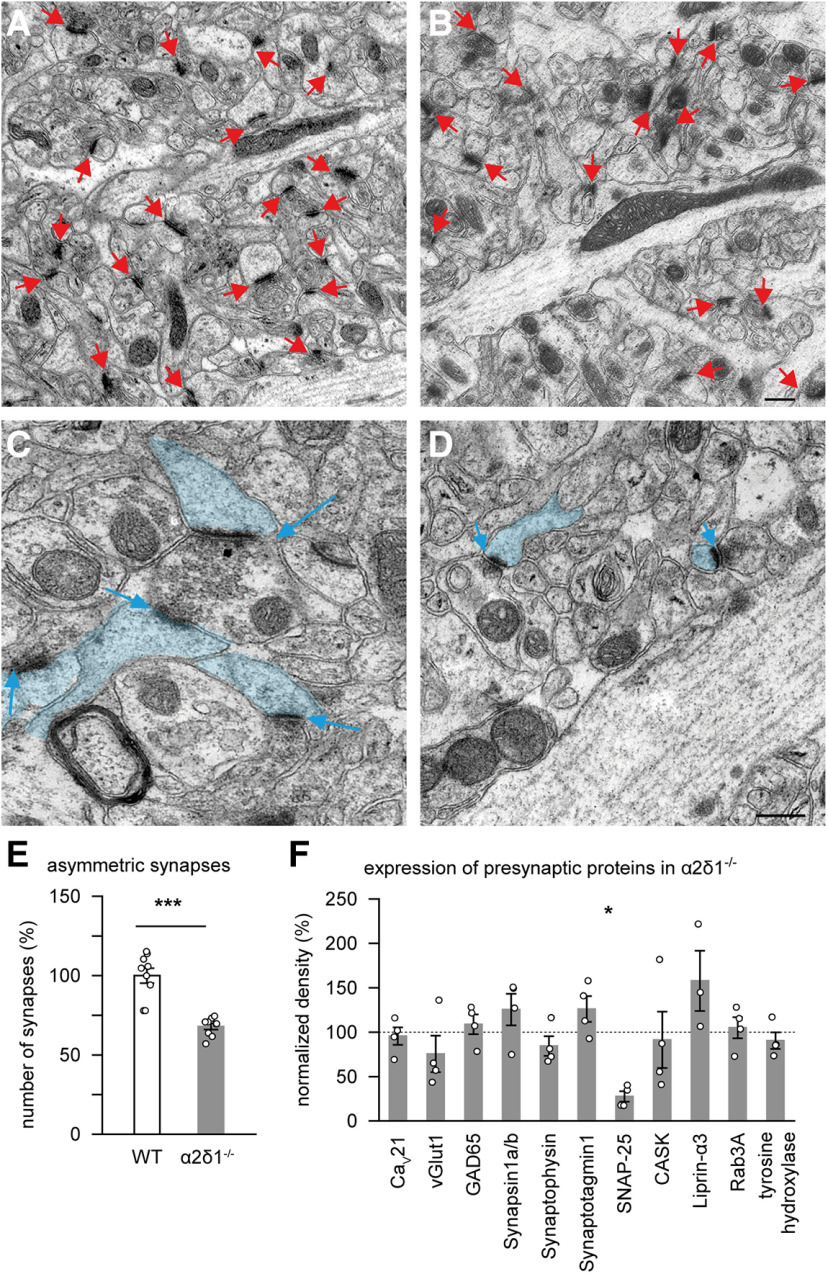
Constitutive KO of the α2δ1 subunit of calcium channels results in a smaller number of asymmetric synapses in the CA1 area of the hippocampus. ***A***, ***B***, Representative areas of panorama images of the CA1 area from WT (***A***) and α2δ1^−/−^ (***B***) mice. Red arrows point to identified asymmetric (presumably excitatory) synapses. Scale bar, 250 nm. ***C***, ***D***, Representative spinous synapses from CA1 of WT (***C***) and α2δ1^−/−^ (***D***) mice. Blue represents postsynaptic spines. Scale bar, 250 nm. ***E***, The mean number of synapses is significantly lower in α2δ1^−/−^ mice (68.0 ± 1.9%, *n* = 9 images) compared with WT controls (100.0 ± 4.7%, *n* = 9 images). ***F***, The constitutive KO of the α2δ1 subunit generally does not alter presynaptic protein composition in α2δ1^−/−^ mice, compared with WT animals. VGlut1, vesicular glutamate transporter 1, GAD65, glutamic acid decarboxylase isoform 65, SNAP-25, synaptosome-associated protein 25 kDa, CASK, calcium/Calmodulin-dependent serine protein kinase, Rab3A, Ras-related protein Rab-3A. *p < 0.05, ***p < 0.001. Means and *n* values are given in Extended Data Figure 1-1.

Since α2δ1 and α2δ3 subunits are both abundant in the hippocampus *in vivo* and in cultured neurons (Klugbauer et al., 1999; Cole et al., 2005; Schlick et al., 2010), we chose to characterize their functional effects on network activity and connectivity in hippocampal neurons as a standard model preparation. Investigations of constitutive α2δ1 KO mice have shown that the chronic loss of α2δ1 subunits has massive impact on structure and density of synapses at least in the cortex (Risher et al., 2018). To first examine whether hippocampal glutamatergic synapses also undergo changes in the constitutive KO model of the α2δ1 subunit, we used transmission electron microscopy. We found changes in both numbers and spine morphology of asymmetric (presumably excitatory) synapses (Fig. 1*A–D*), with synapse density being reduced by 32% compared with WT (Fig. 1*E*). Quantitative immunoblotting of brain lysates from WT and α2δ1^−/−^ mice demonstrated that deletion of the α2δ1 subunit generally did not alter the overall expression levels of various presynaptic marker proteins, including the pore-forming subunit of Ca_V_2.1 channels (Fig. 1*F*). These results confirm and extend recently reported alterations of cortical synapses in the same KO mouse model (Risher et al., 2018). However, the constitutive KOs of α2δ isoforms are associated with severe phenotypes (Striessnig and Koschak, 2008), such as diabetes in the α2δ1 KO mice (Felsted et al., 2017), which might obscure more specific α2δ functions and complicate the distinction between direct and compensatory effects. To brace against this possibility and to be able to alter expression of α2δ1 and α2δ3 at defined time points during development, we mostly used lentivirus-mediated overexpression and knockdown to address the role of these auxiliary subunits in defining the connectivity of neuronal networks.

10.1523/JNEUROSCI.1707-19.2020.f1-1 Figure 1-1 Figure 1 E. The mean number of asymmetric synapses in the CA1 area of the hippocampus (data were normalized to the mean value (taken as 100%) obtained from wild type mice). Figure 1 F. Presynaptic protein composition in α2δ1-/- mice (data were normalized to respective mean values (taken as 100%) in wild type animals). Download Figure 1-1, DOCX file

### α2δ1 and α2δ3 affect neuronal network activity in distinct developmental windows

**Figure 2.**
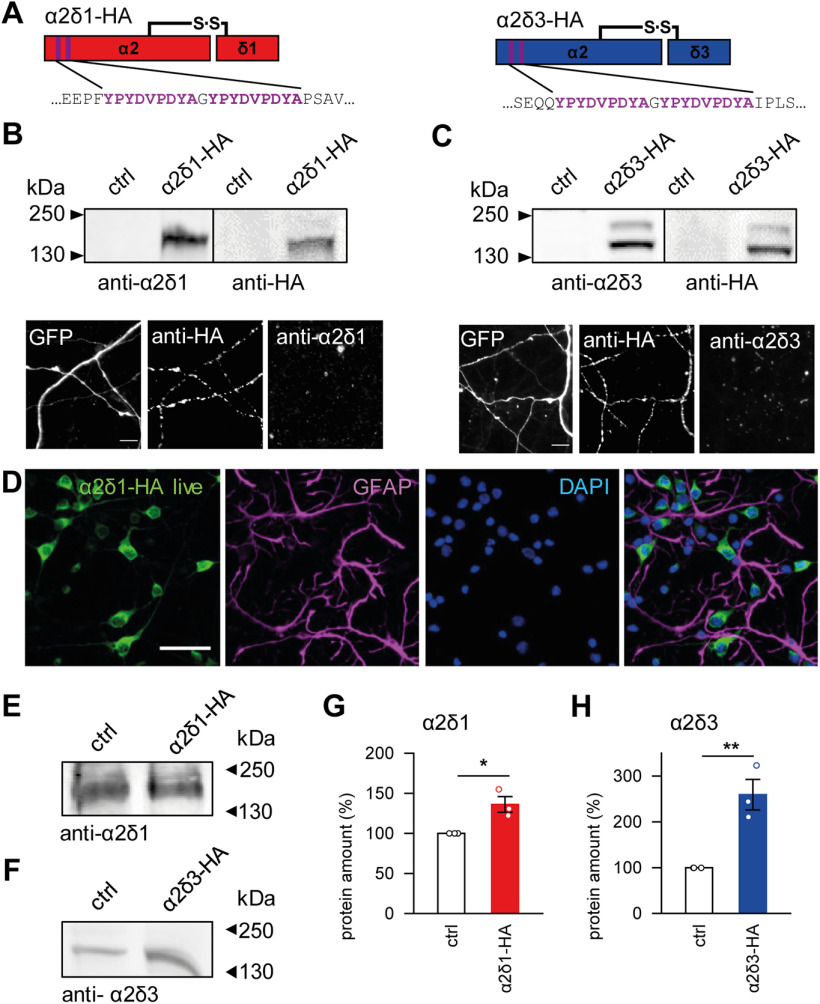
Characterization of HA-tagged α2δ1 and α2δ3 subunits and protein expression levels before and after lentiviral-induced overexpression. ***A***, Schemes of double HA-tagged α2δ1 (left) and α2δ3 (right) subunits. Purple represents the localization of the HA tag. ***B***, ***C***, Validation of the α2δ1 antibodies in Western blots of either untreated HEK293T cells (control, ctrl) or HEK293T cells expressing the α2δ1-HA (***A***) or α2δ3-HA (***B***) subunit. The HA-tagged α2δ proteins were detected using either the anti-α2δ antibodies (left) or a highly specific anti-HA antibody that served as positive control (right). Validation of the α2δ antibodies in live immunocytochemical stainings of DIV16 hippocampal cultures expressing the HA-tagged α2δ subunits and GFP to identify transfected cells. Scale bars, 5 µm. ***D***, Representative images of neuronal cultures at DIV16 stained against the HA tag (live, green), GFAP (magenta), and DAPI (blue) to show α2δ1-HA-infected neurons, glial cells, and the total cell number, respectively. Glial cells do not express the α2δ1 subunit, thus confirming neuron-specific expression. Scale bar, 50 μm. ***E***, ***F***, Exemplary Western blots showing the endogenous (ctrl) and viral-boosted expression of α2δ1 (***E***) or α2δ3 (***F***) in neurons at DIV16. ***G***, ***H***, Lentiviral infection significantly increases total protein level of the α2δ1 (***G***) and α2δ3 (***H***) subunits. GFAP, glial fibrillary acidic protein, DAPI, 4′,6-diamidino-2-phenylindole. *p < 0.05, **p < 0.01. Means and *n* values are given in Extended Data Figure 2-1.

To address the central question whether α2δ1 and/or α2δ3 affect synaptogenesis differently and may interfere with the balance between excitation and inhibition, we infected rat hippocampal cultures with lentiviral particles carrying HA-tagged α2δ1 or α2δ3 subunits. The HA-tag was introduced shortly after the N-terminus of the protein (Fig. 2*A*). The expression, surface delivery (Fig. 2*B-D*) and impact of tagged α2δ subunits on current properties Ca_V_2.1 and Ca_V_2.2 channels were tested. Tagged α2δ1 or α2δ3 subunits had no impact on the current density or voltage-dependent inactivation of channels tested by expression of Ca_V_2.1 or Ca_V_2.2 with the β3 subunit and tagged or untagged α2δ subunits in HEK293T cells (current density: α2δ1, HA 37.2 ± 12.4 pA/pF, *n* = 14; nontagged 33.0 ± 8.9, *n* = 15; α2δ3, HA 58.0 ± 15.3, *n* = 19; nontagged 57.4 ± 14.7, *n* = 16), or Ca_V_2.2 (α2δ1, HA 27.7 ± 7.3 pA/pF, *n* = 11; nontagged 31.0 ± 3.8, *n* = 10; α2δ3, HA 140.3 ± 27.7, *n* = 12; nontagged 115.0 ± 21.5, *n* = 13; half-maximal steady-state inactivation of Ca_V_2.1: α2δ1, HA −26.6 ± 2.3 mV, *n* = 9; nontagged −30.7 ± 3.1 mV, *n* = 13; α2δ3, HA −20.8 ± 1.7 mV, *n* = 13; nontagged −24.0 ± 1.3 mV, *n* = 15; Ca_V_2.2: α2δ1, HA −44.0 ± 1.7 mV, *n* = 12; nontagged −44.7 ± 1.5 mV, *n* = 12; α2δ3, HA −37.0 ± 2.3 mV, *n* = 12; nontagged −37.4 ± 1.2 mV, *n* = 12). Antibodies against α2δ1 or α2δ3 subunits were suitable for biochemical detection of the proteins in Western blot analysis, but not for evaluation of the surface expression of α2δ subunits in live immunocytochemical experiments (Fig. 2*B*,*C*,*E*,*G*). Comparison of the α2δ protein levels in infected cultures to the endogenous level in control sister cultures revealed that total expression of α2δ1 or α2δ3 was significantly increased by 36% or 160%, respectively (Fig. 2*E–G*). These evaluating experiments encouraged us to use the viral expression of the α2δ subunits to probe whether they have a specific impact on neuronal network development and activity.

10.1523/JNEUROSCI.1707-19.2020.f2-1 Figure 2-1 Figure 2 G. Total protein amount of the α2δ1 subunit in neurons (data were normalized to values in non-infected sister controls taken as 100%). Figure 2 H. Total protein amount of the α2δ3 subunit in neurons (data were normalized to values in non-infected sister controls taken as 100%). Download Figure 2-1, DOCX file

**Figure 3.**
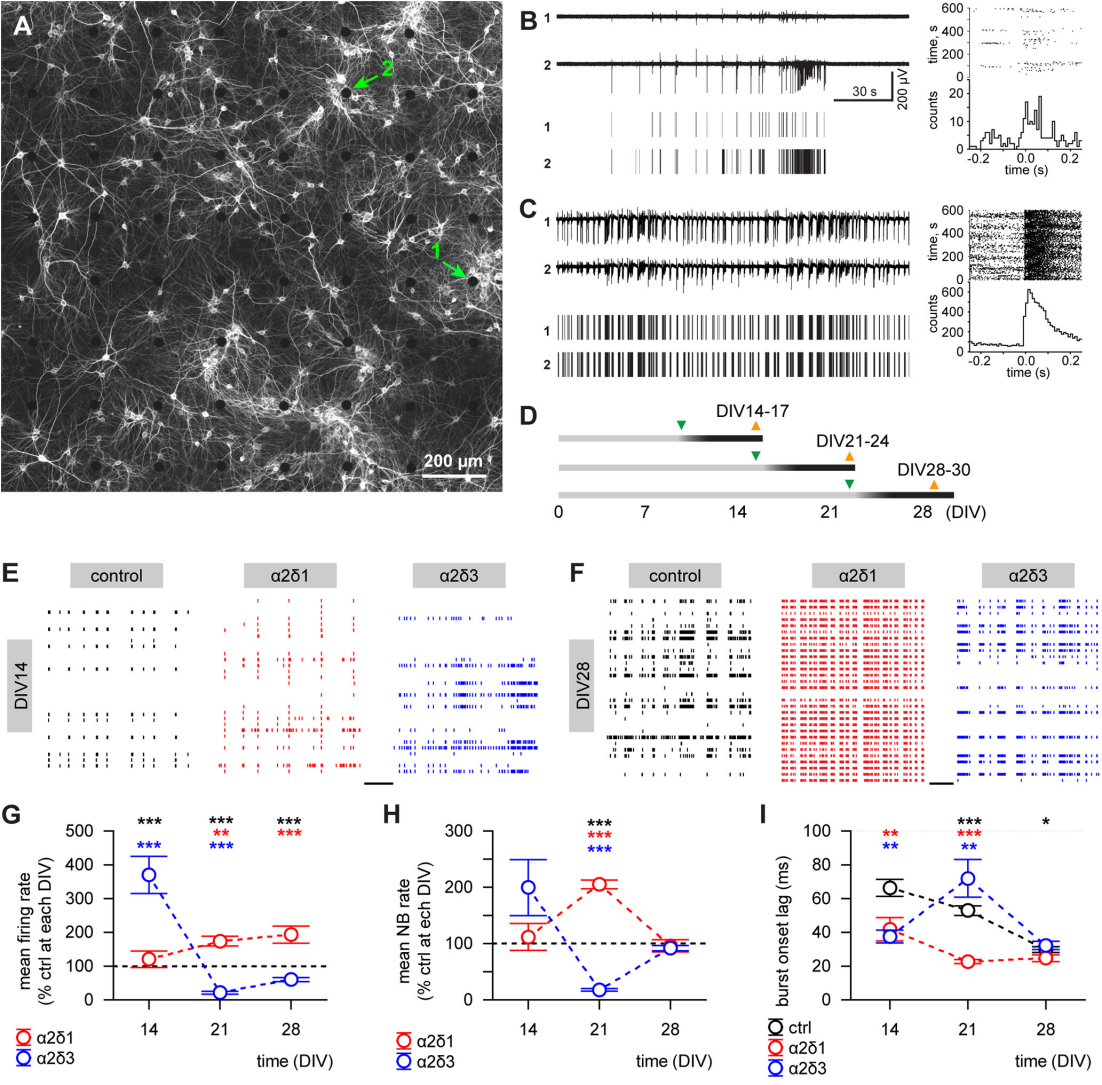
Upregulation of α2δ subunits strongly affects the neuronal network activity in age-dependent manner. ***A***, An example of rat hippocampal culture grown on 60-channel MEA (MAP2 immunostaining of naive mature culture at DIV35). ***B***, ***C***, Traces of activity (top) and corresponding raster representation of detected spikes (bottom) for the same two channels at DIV15 (***B***) and DIV35 (***C***, recorded immediately before immunostaining shown in ***A***). Right, Peristimulus histogram for spikes with channel 1 taken as a trigger. The location of electrodes 1 and 2 is indicated by arrows in ***A***. Note the difference in scaling for spike counts. ***D***, A timeline of infection (green triangle) and recording (orange triangle) in different cohorts of rat hippocampal cultures infected during second (recorded after DIV14; control *n* = 6 MEAs, α2δ1 *n* = 5, α2δ3 *n* = 5), third (recorded after DIV21; all groups *n* = 8), or fourth (recorded after DIV28; control *n* = 10, α2δ1 *n* = 7, α2δ3 *n* = 6) weeks *in vitro*. ***E***, ***F***, Representative raster plots of spontaneous neuronal network activity recorded in developing (***E***, DIV14; scale bar, 60 s) and mature(***F***; DIV28; scale bar, 10 s) cultures. Thirty of 60 channels from each array are shown. ***G***, Overexpression of the α2δ3 subunit strongly enhances the mean firing rate at DIV14 but suppresses it at DIV21, whereas the α2δ1 upregulation-induced enhancement of neuronal activity is evident later in development (DIV21-DIV28). ***H***, Overexpression of α2δ1 or α2δ3 subunits during the third week *in vitro* is associated with opposite effects on functional network interaction at DIV21. ***I***, Overexpression of α2δ3 improves synchronization of bursting activity across the network during, but not after, the second developmental week, whereas upregulation of α2δ1 consistently decreases the burst onset lag. *p < 0.05, **p < 0.01, ***p < 0.001. Means and *n* values are given in Extended Data Figure 3-1.

Because of the default absence of external inputs, the development of cultured neuronal networks is rather stereotypical and culminates in developmental arrest on maturation after ∼28 DIV (van Pelt et al., 2004; Bettencourt et al., 2007; Bikbaev et al., 2015). As a consequence, the spontaneous network activity emerging in neuronal cultures faithfully reflects solely intrinsic formation and maturation of the network connectivity (Fig. 3*A–C*) without being influenced or masked by external sensory inputs. Therefore, three cohorts of cultures grown on 60-channel MEAs were infected after 7, 14, or 21 DIV, and the spontaneous activity was recorded ∼1 week after infection (Fig. 3*D*). We found that upregulation of α2δ subunits differentially affected the mean firing rate. Depending on the infection time point, α2δ1 and α2δ3 subunits showed opposite (all *p* < 0.001, one-way ANOVA; Fig. 3*E-I*) effects. Upregulation of α2δ3 during second developmental week increased the neuronal firing almost fourfold by DIV14 compared with age-matched control or α2δ1-overexpressing cultures (both *p* < 0.001, Duncan's test; Fig. 3*E*,*G*). In contrast, α2δ3 overexpression after DIV14 strongly suppressed neuronal firing to 21 ± 4% by DIV21, compared with the mean values in controls (*p* < 0.001, Duncan's test). Overexpression of α2δ1 had no impact by DIV14 but consistently increased the mean firing rate after DIV14 compared with corresponding values in controls or α2δ3-overexpressing cultures at DIV21 (*p* < 0.01 and *p* < 0.001, respectively; Duncan's test), with the difference being even more pronounced at DIV28 (both *p* < 0.001, Duncan's test; Fig. 3*F*,*G*).

10.1523/JNEUROSCI.1707-19.2020.f3-1 Figure 3-1 Figure 3 G. Mean spontaneous firing rate in hippocampal cultures grown on MEAs (normalized for each culture to the mean value in control group at respective DIV). Figure 3 H. Mean network burst (NB) rate per min in hippocampal cultures overexpressing the α2δ subunits (normalized for each culture to the mean value in control group at respective DIV). Figure 3 I. Mean burst onset lag in hippocampal cultures overexpressing the α2δ subunits. Download Figure 3-1, DOCX file

Next, we examined the impact of α2δ overexpression on the functional connectivity. For this purpose, we analyzed the occurrence rate of NBs and the burst onset lag, which reflect episodes of functional network interaction between remote neuronal clusters and synchronization of their bursting activity across the network (Bikbaev et al., 2015). At DIV14, we observed no significant change in the mean NB rate on upregulation of α2δ subunits. Intriguingly, functional network interaction at DIV21 was strongly enhanced on α2δ1 overexpression, whereas upregulation of the α2δ3 subunit led to dramatic suppression of NBs (Fig. 3*H*). Remarkably, the effect of α2δ3 upregulation on the synchronicity of the bursting onset was reversed during the third week *in vitro*: the burst onset lag was shorter at DIV14, but longer at DIV21 in comparison with respectivevalues in age-matched controls (both *p* < 0.01, Duncan's test; Fig. 3*I*). In α2δ1-overexpressing cultures, the burst onset lag was shorter than in controls at DIV14 and DIV21 (*p* < 0.01 and *p* < 0.001, respectively; Duncan's test).

Thus, we found that overexpression of α2δ1 and α2δ3 differentially changes the spontaneous neuronal firing and network interaction in a development-dependent manner. These results indicate that upregulation of the α2δ subunits indeed alters the excitatory-to-inhibitory balance in developing hippocampal networks and raise the question how upregulation of α2δ1 and α2δ3 affects the transmission in excitatory and inhibitory synapses.

### α2δ1 subunit selectively enhances presynaptic release in excitatory and α2δ3 in inhibitory synapses

**Figure 4.**
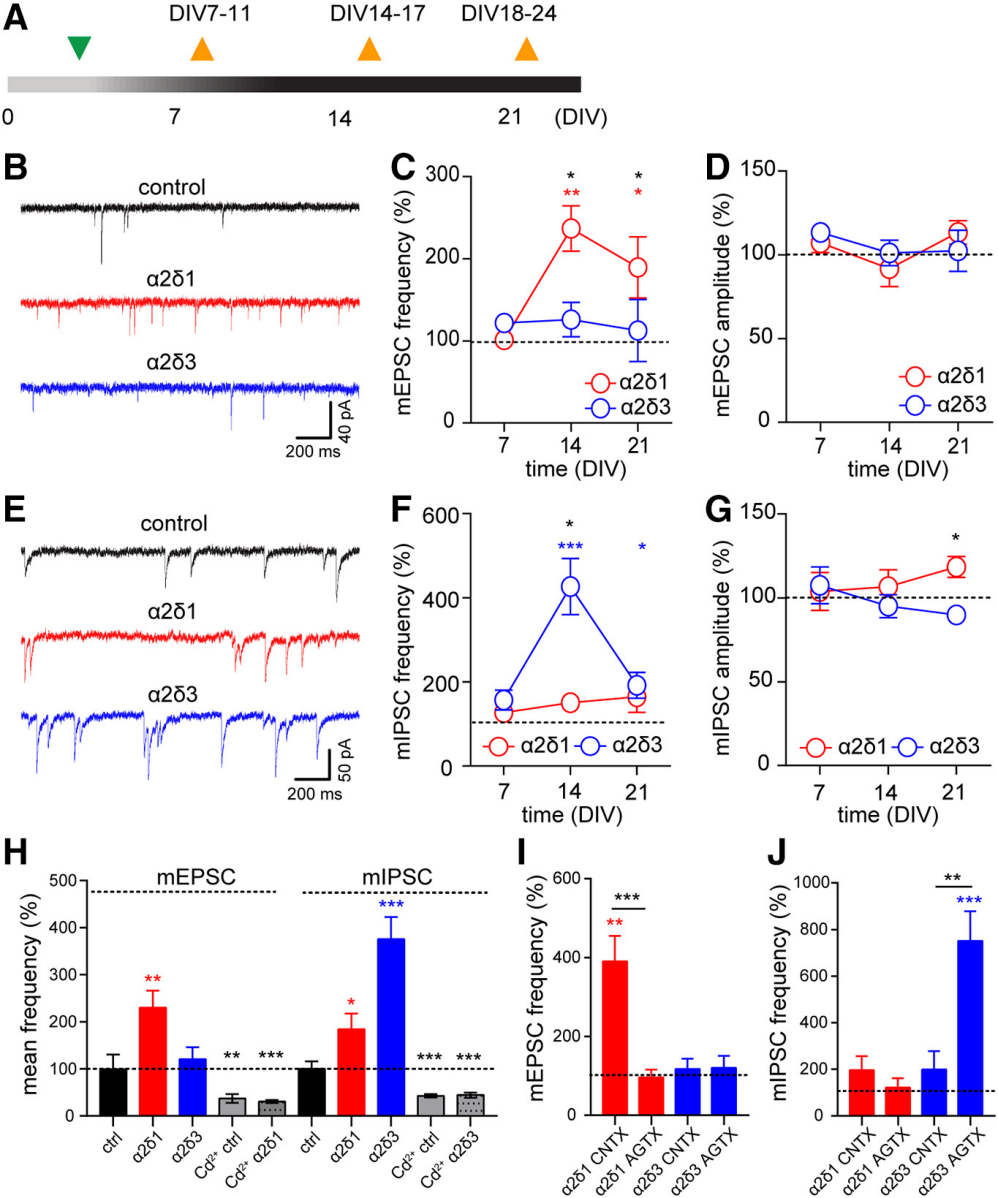
Overexpression of α2δ1 and α2δ3 subunits selectively increases the frequency of neurotransmitter release in excitatory and inhibitory synapses, respectively. ***A***, A timeline of infection (green triangle) and electrophysiological recordings (orange triangles). ***B***, Representative traces of mEPSCs recorded at DIV14 in control and α2δ1- and α2δ3-overexpressing cultures. ***C***, ***D***, The mean frequency (***C***) and the amplitude (***D***) of mEPSCs in α2δ1- and α2δ3-overexpressing cultures. ***E***, Representative traces of mIPSCs recorded at DIV14 in control and α2δ1- and α2δ3-overexpressing cultures. ***F***, ***G***, The mean frequency (***F***) and the amplitude (***G***) of mIPSCs in α2δ1- and α2δ3-overexpressing cultures. ***H***, The increase in the mEPSC and mIPSC frequency by α2δ1 and a2d3 subunits, respectively, is caused by bigger contribution of high voltage-activated VGCCs as demonstrated by Cd^2+^-induced reduction to respective values obtained in controls in the presence of Cd^2+^. ***I***, ***J***, The effects of α2δ1 and α2δ3 overexpression on the frequency of mEPSCs (***E***) and mIPSCs (***F***) are mediated by P/Q- and N-type calcium channels, respectively. CNTX, conotoxin, AGTX, agatoxin. *p < 0.05, **p < 0.01, ***p < 0.001. Means and *n* values are given in Extended Data Figure 4-1.

The enhancement of neuronal firing in α2δ1-overexpressing cultures (Fig. 3*G*) could potentially reflect a reported earlier increase in glutamate release and synapse structure (Hoppa et al., 2012; Schneider et al., 2015) or be caused by a decreased release of GABA. Similarly, the α2δ3-induced suppression of the network activity after DIV14 indicated a shift inthe excitatory-to-inhibitory balance due to either enhanced GABA release or reduced release of glutamate. To clarify this, we measured mEPSCs and mIPSCs in neurons overexpressing either α2δ1 or α2δ3 subunits. To enable recordings in developing neurons, in the following experiments, the primary hippocampal cultures were infected during first developmental week at DIV2-DIV4. Subsequently, mEPSCs and mIPSCs were recorded in the presence of TTX, APV, and either DNQX or bicuculline, respectively, at three time points between DIV7 and DIV21 (Fig. 4*A*). No significant effect of α2δ upregulation on miniature currents was observed in 1-week-old cultures. At DIV14 and DIV21, the mean mEPSC frequency was higher in cultures overexpressing the α2δ1, but not α2δ3, compared with control values at corresponding time points (*p* < 0.01 and *p* < 0.05, respectively; Dunn's test; Fig. 4*B*,*C*). In striking contrast, upregulation of the α2δ3, but not α2δ1, strongly increased the mean mIPSC frequency at DIV14 and DIV21 compared with age-matched controls (*p* < 0.001 and *p* < 0.05, respectively; Dunn's test; Fig. 4*E*,*F*). The amplitude of miniature currents was not affected by α2δ overexpression compared with control values at any time point (Fig. 4*D*,*G*). However, the mIPSC amplitude at DIV21 was significantly smaller in α2δ3-overexpressing cultures compared with cultures overexpressing the α2δ1 subunit (*p* < 0.05; Dunn's test; Fig. 4*G*).

10.1523/JNEUROSCI.1707-19.2020.f4-1 Figure 4-1 Figure 4 C. Mean mEPSC frequency (normalized to control mean at respective DIV). Figure 4 D. Mean mEPSC amplitude (normalized to control mean at respective DIV). Figure 4 F. Mean mIPSC frequency (normalized to control mean at respective DIV). Figure 4 G. Mean mIPSC amplitude (normalized to control mean at respective DIV). Figure 4 H. Mean mEPSC and mIPSC frequency (normalized to corresponding mean value in respective control groups). Figure 4 I. Mean mEPSC frequency (normalized to control mean value with respective toxin). Figure 4 J. Mean mIPSC frequency (normalized to control mean value with respective toxin). Download Figure 4-1, DOCX file

The stochastic opening of high VGCCs accounts for ∼50% of mEPSCs and mIPSCs (Goswami et al., 2012; Williams et al., 2012; Ermolyuk et al., 2013). Therefore, the pronounced effect of α2δ overexpression on the mEPSCs and mIPSCs after DIV14 (Fig. 4*C*,*F*) strongly suggested a bigger contribution of VGCCs to spontaneous release. Indeed, we found that acute blockade of VGCCs by cadmium (Cd^2+^) strongly decreased the frequency of miniature currents in 2-week-old cultures overexpressing the α2δ1 (mEPSCs: *p* < 0.001, Mann–Whitney test) or α2δ3 (mIPSCs: *p* < 0.001) subunit to respective control levels obtained in the presence of Cd^2+^ from noninfected cultures (Fig. 4*H*).

In central synapses, the neurotransmitter release is triggered predominantly by Ca_V_2.1 and Ca_V_2.2 (Wheeler et al., 1994; Scholz and Miller, 1995; Cao and Tsien, 2010), but their abundance at excitatory and inhibitory presynaptic terminals may vary (Iwasaki et al., 2000). To clarify whether the elevation of the mEPSC and mIPSC frequency by α2δ1 and α2δ3 subunits involves distinct subpopulations of presynaptic VGCCs, we performed additional patch-clamp recordings in the presence of isoform-specific channel blockers. In α2δ1-overexpressing cultures, the blockade of Ca_V_2.2 by ω-conotoxin GVIA did not abolish the increase in the mean mEPSC frequency, but the blockade of Ca_V_2.1 by ω-agatoxin IVA reduced the mEPSC frequency (*p* < 0.001, Dunn's test) to a level observed in control cultures treated with agatoxin (Fig. 4*I*). In contrast, we found that the α2δ3 overexpression-induced increase in mIPSC frequency was abolished by conotoxin (*p* < 0.01, Dunn's test), but not by agatoxin (Fig. 4*J*), compared with control values obtained in the presence of respective toxins.

These results revealed a selective impact of the α2δ1and α2δ3 calcium channel subunits on the spontaneous neurotransmitter release in excitatory and inhibitory synapses. Furthermore, we found that facilitation of the spontaneous glutamate release by α2δ1 is predominantly mediated by Ca_V_2.1, whereas α2δ3-driven enhancementof GABA release involved mainly Ca_V_2.2 calcium channels.

### shRNA-mediated knockdown of α2δ1 and α2δ3 subunits mirror the effects of overexpression on neurotransmitter release and network activity

To rule out possible artifacts of overexpression or lentiviral infection and verify that the effects on neurotransmitter release and the neuronal firing are caused by the overexpression of α2δ subunits, we acutely knocked down the α2δ1 and α2δ3 subunits using specific shRNAs.

**Figure 5.**
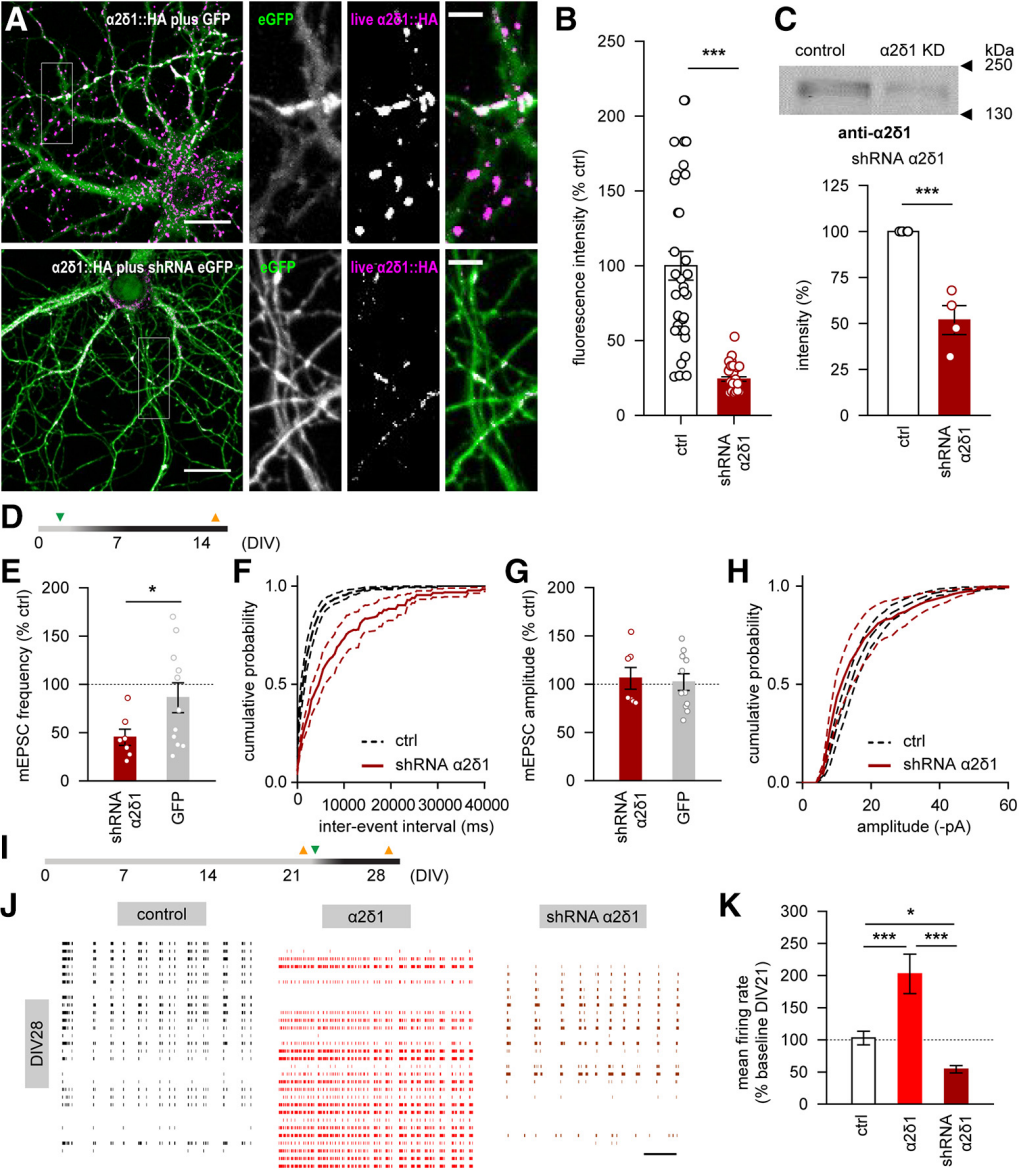
Downregulation of the α2δ1 subunit impairs the presynaptic release of glutamate and abolishes α2δ1 overexpression-driven enhancement of spontaneous neuronal firing. ***A***, ***B***, shRNA-induced knockdown of the α2δ1 subunit results in significant decrease of its surface expression and in corresponding decrease of the live HA fluorescence in rat hippocampal neurons. ***C***, Western blot demonstrates a significant decrease in neuronal expression of the α2δ1 subunit on shRNA-triggered knockdown. ***D***, A timeline of infection (green triangle) and electrophysiological recordings (orange triangles) shown in ***E–H***. ***E***, Downregulation of the α2δ1 subunit, but not the GFP expression, leads to significant reduction of the mean frequency of mEPSCs in rat hippocampal neurons. ***F***, Cumulative distribution of interevent intervals for mEPSCs recorded under control conditions or on α2δ1 knockdown. ***G***, The mean mEPSC amplitude is not affected by either α2δ1 knockdown, or by lentiviral expression of the GFP. ***H***, Cumulative distribution of mEPSC amplitudes recorded under control conditions or on α2δ1 knockdown. ***I***, A timeline of infection (green triangle) and multichannel recordings (orange triangles) shown in ***J***, ***K***. ***J***, Representative traces of spontaneous neuronal firing in rat hippocampal cultures under control conditions (black), as well as after 1 week of either α2δ1 overexpression (red) or knockdown (brown). Thirty of 60 channels from each array are shown. Scale bar, 10 s. ***K***, The shRNA-mediated knockdown of the α2δ1 subunit during the fourth week *in vitro* is associated with suppression of the spontaneous neuronal firing. *p < 0.05, ***p < 0.001. Means and *n* values are given in Extended Data Figure 5-1.

For the α2δ1 subunit, both live anti-HA labeling of HA-tagged α2δ1 subunits and Western blot analysisof the total α2δ1subunit population demonstrated strong downregulation in neurons (Fig. 5*A–C*). Since the most pronounced effect of the α2δ1 overexpression on the glutamate release was observed at DIV14 (Fig. 4*B*,*C*), we recorded mEPSCs in neurons at DIV14 on α2δ1 downregulation, as well as in neurons infected with GFP-expressing lentiviral particles that served as lentiviral infection control (Fig. 5*D*). We found that shRNA-induced α2δ1 knockdown markedly reduced the mEPSC frequency compared with noninfected controls (*p* < 0.05, Mann–Whitney test; Fig. 5*E*,*F*). No effect of lentiviral expression of GFP on the mEPSC frequency or amplitude was found (Fig. 5*E–H*).

10.1523/JNEUROSCI.1707-19.2020.f5-1 Figure 5-1 Figure 5 B. Mean live HA fluorescence intensity (normalized to mean values in sister control cultures) Figure 5 C. Levels of α2δ1 expression (normalized to mean values in sister control cultures). Figure 5 E. Mean mEPSC frequency in hippocampal neurons (normalized to the mean value in respective controls). Figure 5 G. Mean mEPSC amplitude in hippocampal neurons (normalized to the mean value in respective controls). Figure 5 K. Mean spontaneous firing rate at DIV28 one week after lentiviral infection in hippocampal cultures grown on MEAs (control n=10 MEAs; α2δ1 overexpression n=6 MEAs; α2δ1 knock-down n=5 MEAs) (normalized for each culture to own pre-infection mean value at DIV21 (taken as 100%)). Download Figure 5-1, DOCX file

Since the strongest effect of α2δ1 overexpression on the network activity was observed at DIV28 (Fig. 3*G*), in an additional set of 3-week-old cultures, we induced upregulation or downregulation of the α2δ1 subunit and assessed spontaneous neuronal firing 1 week later (Fig. 5*I*). In control cultures, no significant change of the firing rate was observed between DIV21 and DIV28. The upregulation of α2δ1 enhanced neuronal firing (*p* < 0.001, Duncan's test), whereas the α2δ1 knockdown led to suppression of the mean firing rate compared with values in control and α2δ1-overexpressing cultures (*p* < 0.05 and *p* < 0.001, respectively; Duncan's test; Fig. 5*J*,*K*).

**Figure 6.**
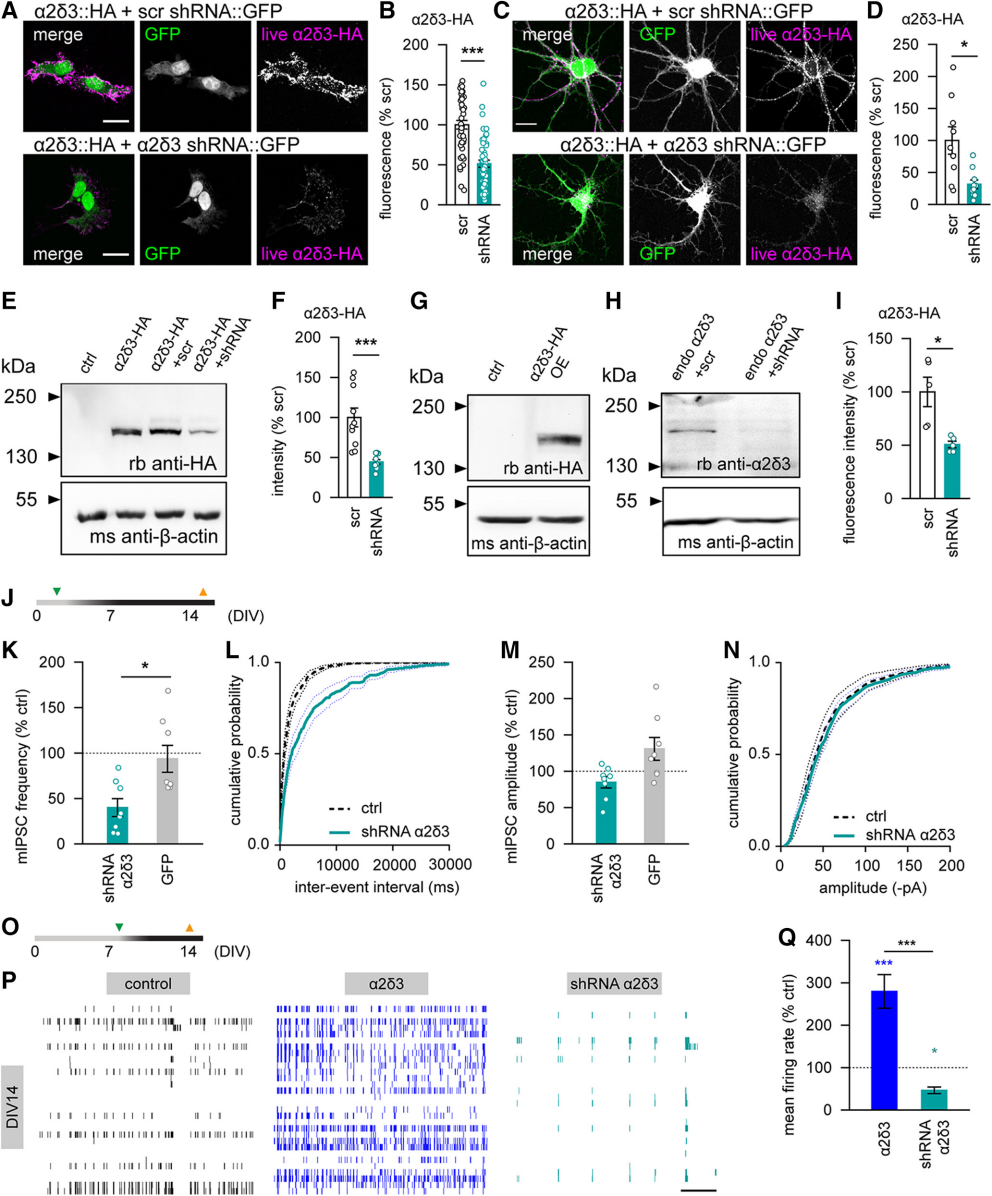
Downregulation of the α2δ3 subunit impairs spontaneous GABA release and leads to suppression of neuronal firing in developing hippocampal neurons. ***A***, ***B***, The shRNA-mediated knockdown of the α2δ3 subunit results in a significant decrease of the α2δ3 surface expression in HEK293T was examined via fluorescence labeling of HA-tagged α2δ3 subunits in HEK293T cells (***A***,***B***), compared with the effect of scrambled (scr) shRNA. Scale bar, 20 µm. ***C***, ***D***, Downregulation of the α2δ3 subunit in rat hippocampal neurons. Scale bar, 20 µm. ***E***, ***F***, Western blots of HEK293T cells expressing the HA-tagged α2δ3 subunit together with the scrambled shRNA control or the α2δ3 shRNA. ***G-I***, Western blots of hippocampal cultures infected with the HA-tagged α2δ3 construct (***G***) or with the scrambled shRNA control, as well as the α2δ3 shRNA (blue) (***H***,***I***). ***J***, A timeline of infection (green triangle) and electrophysiological recordings (orange triangles) shown in ***K***, ***N***. ***K***, Downregulation of the α2δ3 subunit, but not the GFP expression, significantly decreases the mean mIPSC frequency in rat hippocampal neurons. ***L***. Cumulative distribution of interevent intervals for mEPSCs recorded under control conditions or on α2δ1 knockdown. ***M***, The mean mEPSC amplitude is not affected by either α2δ3 knockdown or by lentiviral expression of the GFP. ***N***, Cumulative distribution of mEPSC amplitudes recorded under control conditions or on α2δ1 knockdown. ***O***, A timeline of infection (green triangle) and multichannel recordings (orange triangles) shown in ***P***, ***Q***. ***P***, Representative traces of spontaneous neuronal firing in rat hippocampal cultures under control conditions (black), as well as after 1 week of either α2δ3 overexpression (blue) or α2δ3 knockdown (petrol). Thirty of 60 channels from each array are shown. Scale bar, 20 s. ***Q***, The α2δ3 overexpression in young neurons strongly enhances neuronal activity, whereas the shRNA-mediated α2δ3 knockdown leads to dramatic suppression of the mean firing rate. *p < 0.05, ***p < 0.001. Means and *n* values are given in Extended Data Figure 6-1.

Similar experiments were conducted using shRNA constructs to knock down the α2δ3 subunit. Evaluation of the construct demonstrated a robust suppression of α2δ3 subunit expression by 50%–60% in HEK cells (*p* < 0.001, Mann–Whitney test; Fig. 6*A*,*B*) and primary hippocampal cultured neurons (*p* < 0.05; Fig. 6*C*,*D*). Furthermore, the quantification of the α2δ3 expression level in neuronal cultures demonstrated a significant reduction on shRNA-mediated knockdown both in HEK cells (*p* < 0.001; Fig. 6*E*,*F*) and in neurons (*p* < 0.05; Fig. 6*G–I*).

10.1523/JNEUROSCI.1707-19.2020.f6-1 Figure 6-1 Figure 6 B. Live fluorescence intensity of HA-tagged α2δ3 subunits in HEK293T cells (data were normalized to the mean in cells expressing α2δ3-HA with scrambled shRNA). Figure 6 D. Live fluorescence intensity of α2δ3-HA in hippocampal neurons at DIV9 (data were normalized to the mean in neurons expressing α2δ3-HA with scrambled shRNA). Figure 6 F. Western blots of HEK293T cells expressing the HA-tagged α2δ3 subunit together with the scrambled shRNA or the α2δ3 shRNA (data were normalized to the mean in preparations with scrambled shRNA). Figure 6 I. Western blots of hippocampal cultures expressing the HA-tagged α2δ3 subunit together with the scrambled shRNA or the α2δ3 shRNA (data were normalized to the mean in preparations with scrambled shRNA). Figure 6 K. Mean mIPSC frequency in hippocampal neurons (normalized to the mean value in respective controls). Figure 6 M. Mean mIPSC amplitude in hippocampal neurons (normalized to the mean value in respective controls). Figure 6 Q. Mean spontaneous firing rate at DIV14 one week after lentiviral infection in hippocampal cultures grown on MEAs (control n=7 MEAs; α2δ3 overexpression n=5 MEAs; α2δ3 knock-down n=4 MEAs) (normalized to the mean value in controls). Download Figure 6-1, DOCX file

Functional analysis of the α2δ3 knockdown demonstrated that higher frequency of spontaneous GABA release and the enhance neuronal network activity in young α2δ3-overexpressing cultures were indeed caused by upregulation of this auxiliary subunit. We found that the frequency of mIPSCs was markedly decreased on α2δ3 knockdown, but not GFP expression, compared with controls (*p* < 0.05, Mann–Whitney test; Fig. 6*J–L*). The amplitudes of mIPSCs where not affected in any of the groups (Fig. 6*M*,*N*). Finally, a comparison of the spontaneousactivity recorded under control conditions or on α2δ3 upregulation or downregulation (Fig. 6*O*) revealed that shRNA-mediated α2δ3 knockdown resulted in suppression of spontaneous neuronal firing compared with values in control or α2δ3-overexpressing cultures (*p* < 0.05 and *p* < 0.001, Duncan's test; Fig. 6*P*,*Q*).

So far, these data revealed a selective impact of the α2δ1 as well as α2δ3 calcium channel subunit on the presynaptic neurotransmitter release in excitatory and inhibitory synapses. Given these findings, next we asked whether the elevated frequency of miniature currents on upregulation of α2δ subunits reflects corresponding changes in the number of glutamatergic and/or GABAergic synaptic contacts.

### Upregulation of α2δ3 subunit selectively promotes inhibitory synaptogenesis

**Figure 7.**
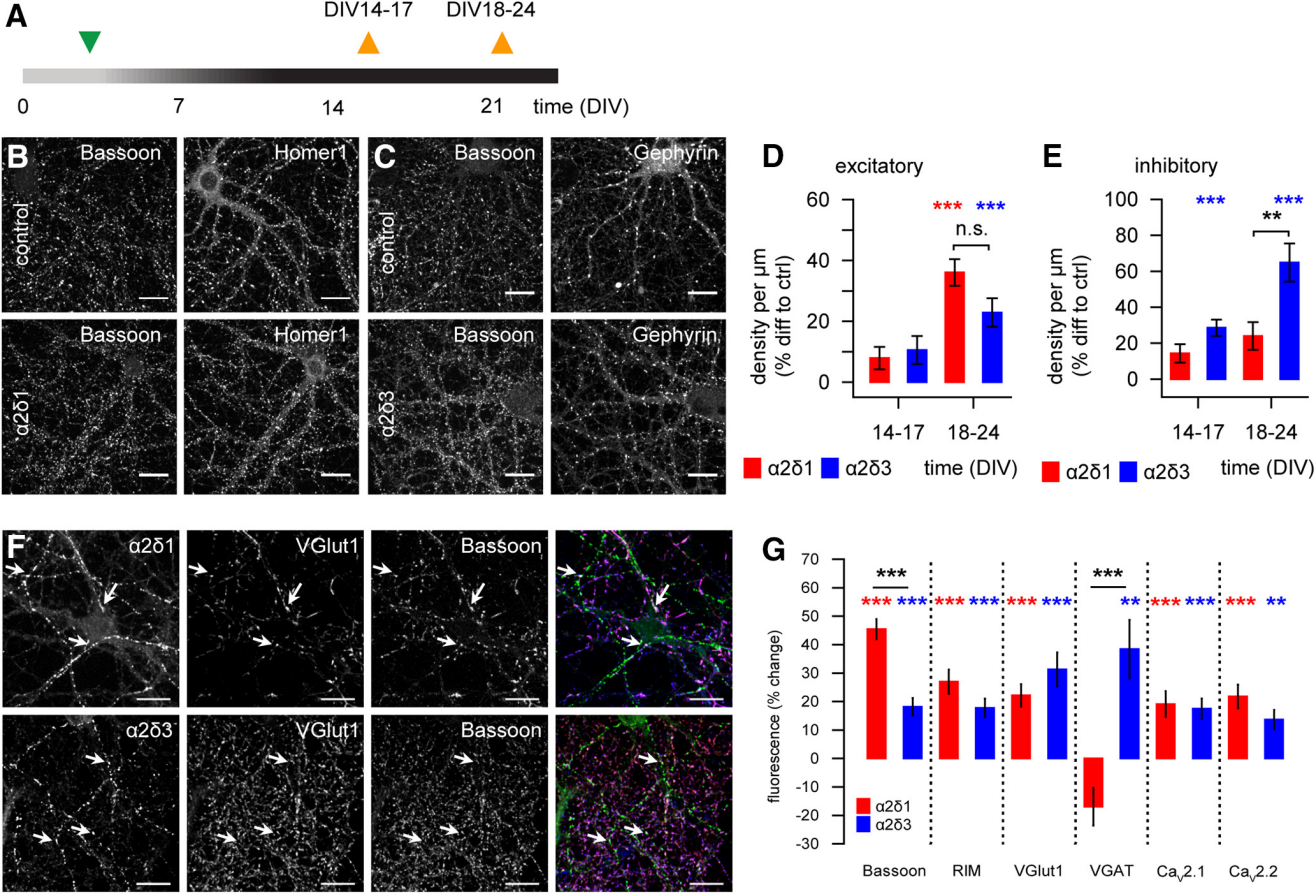
Overexpression of α2δ subunits of calcium channels increases the synaptic density in rat hippocampal cultures. ***A***, A timeline of infection (green triangle) and immunolabeling (orange triangles). ***B***, ***C***, Representative images of infected hippocampal cultures (DIV18) stained for either Bassoon and Homer1 (***B***) or Bassoon and Gephyrin (***C***). Scale bars, 20 µm. ***D***, Lentiviral infection-driven overexpression of α2δ1 or α2δ3 in hippocampal cultures increases the number of glutamatergic synapses by the end of the third week *in vitro* compared with controls. ***E***, Upregulation of the α2δ3, but not the α2δ1, subunit results in a marked increase in the density of inhibitory GABAergic synapses already after 2 weeks *in vitro*, compared with respective control values. ***F***, Representative images of transfected hippocampal neurons stained for Bassoon, VGlut1, and HA in either α2δ1- or α2δ3-overexpressing neurons at DIV18-DIV24. Arrows indicate colocalized punctae. Scale bars, 10 µm. ***G***, Mean fluorescence intensity in HA-positive puncta for Bassoon, RIM, VGlut1, VGAT, Ca_V_2.1, and Ca_V_2.2 in transfected rat hippocampal cultures overexpressing either α2δ1 or α2δ3 subunits. RIM, Rab interacting molecule 1/2, VGAT, vesicular GABA transporter. **p < 0.01, ***p < 0.001. Means and *n* values are given in Extended Data Figure 7-1.

The α2δ1 subunit was reported earlier to trigger excitatory synaptogenesis in mouse retinal ganglion cells and cortical neurons (Eroglu et al., 2009), but it remained unknown whether α2δ3 plays a similar role in central synapses. To clarify this, we labeled the presynaptic scaffold protein Bassoon and the postsynaptic scaffold protein Homer1 or Gephyrin to identify glutamatergic and GABAergic synapses, respectively. The immunolabeling was conducted in hippocampal cultures 2-3 weeks after infection at DIV14-DIV24 (Fig. 7*A*). Using colocalization of presynaptic and postsynaptic markers distributed along dendrites (Fig. 7*B*,*C*), we evaluated the density of synaptic contacts per µm (for details, see Materials and Methods).

10.1523/JNEUROSCI.1707-19.2020.f7-1 Figure 7-1 Figure 7 D. Mean number of glutamatergic synapses per μ mm (normalized to the mean values in control sister cultures at respective DIV). Figure 7 E. Mean number of GABAergic synapses per μ mm (normalized to the mean values in control sister cultures at respective DIV). Figure 7 G. Mean fluorescence intensity in HA-positive puncta for presynaptic proteins in transfected rat hippocampal cultures overexpressing either α2δ1, or α2δ3 subunits (data were normalized to respective mean values (taken as 100%) in sister control cultures). Download Figure 7-1, DOCX file

In 2-week-old cultures, we observed a moderate increase of the density of glutamatergic synapses both in α2δ1- and in α2δ3-overexpressing cultures compared with control sister cultures, but the effect was not significant (*p* = 0.17, Kruskal-Wallis ANOVA). After DIV21, the excitatory synapse number was significantly affected by α2δ overexpression (*p* < 0.001, Kruskal-Wallis ANOVA), with the synaptic density being higher in the α2δ1- and in the α2δ3-overexpressing cultures compared with control values (both *p* < 0.001; Fig. 7*D*). These data confirmed the synaptogenic potential of the α2δ1 subunit (Eroglu et al., 2009) but also showed that α2δ3 upregulation can promote excitatory synaptogenesis. More importantly, we found that overexpression of the α2δ3, but not the α2δ1, subunit significantly increased the GABAergic synapse number already at DIV14 compared with control cultures (*p* < 0.001, Dunn's test; Fig. 7*E*). The effect of α2δ3 overexpression on the inhibitory synaptogenesis was even more pronounced in cultures after DIV21, compared with control or α2δ1-overexpressing cultures (*p* < 0.001 and *p* < 0.01, respectively; Dunn's test). Comparison of the fluorescence intensity of presynaptic and postsynaptic scaffolds in excitatory synapses revealed no difference from the control conditions (tested for Bassoon and Homer). Within inhibitory synapses α2δ1 and α2δ3 expression increased the fluorescence intensity of Bassoon in 2- and 3-week-old cultures compared with age-matched controls (DIV14-DIV17: control 100 ± 5% *n* = 69, α2δ1 118 ± 5% *n* = 67, α2δ3 128 ± 5% *n* = 65/3-week-old (DIV18-DIV24: control 100 ± 5% *n* = 67, α2δ1 128 ± 8% *n* = 72, α2δ3 164 ± 11% *n* = 99; *p* < 0.01 and *p* < 0.001, respectively; Kruskal-Wallis ANOVA). The fluorescence of Gephyrin was markedly affected only in 3-week-old cultures (DIV18-DIV24: control 100 ± 4%, α2δ1 92 ± 4%, α2δ3 81 ± 4%; *p* < 0.001 Kruskal-Wallis ANOVA), with values obtained in α2δ3-overexpressing cultures being smaller than in controls (*p* < 0.001, Dunn's test).

The transfection-induced overexpression of α2δ subunits triggers accumulation of presynaptic proteins via increased surface expression of VGCCs (Hoppa et al., 2012; Schneider et al., 2015), which we could not reveal in lentiviral infected cultures. This, in turn, leads to recruitment of presynaptic scaffold components when expressed in combination with the α1 subunit (Davydova et al., 2014; Schneider et al., 2015). To verify that, we assessed the fluorescence intensity of several key presynaptic proteins in hippocampal cultures transfected either with α2δ1-HA or α2δ3-HA. The transfection-induced overexpression allowed us to distinguish the HA-positive transfected synapses and HA-negative puncta of nontransfected neurons embedded into the same network. Indeed, upregulation of either α2δ1 or α2δ3 led to an enhanced accumulation of Bassoon and RIM (Fig. 7*F*,*G*; both *p* < 0.001) that was more pronounced for Bassoon on α2δ1 upregulation (*p* < 0.001, Bonferroni test). Furthermore, the upregulation of α2δ1 or α2δ3 significantly increased the fluorescence of VGlut1, indicating a structural change of excitatory synapses (Fig. 7*G*; both *p* < 0.001). Remarkably, the fluorescence of VGAT, an inhibitory synapse-specific marker, was 38 ± 9% higher only in α2δ3-overexpressing neurons compared with control or α2δ1-overexpressing neurons (*p* < 0.01 and *p* < 0.001, respectively; Fig. 7*G*). Consistent with previous reports (Hoppa et al., 2012; Schneider et al., 2015), upregulation of α2δ1 or α2δ3 subunits increased the synaptic abundance of Ca_V_2.1 (both *p* < 0.001) and Ca_V_2.2 (*p* < 0.001 and *p* < 0.01, respectively; Fig. 7*G*). No differences between α2δ1- or α2δ3-overexpressing neurons in the fluorescence intensity of either Ca_V_2.1 or Ca_V_2.2 were found.

These findings demonstrate that upregulation of α2δ1 or α2δ3 subunits in rat hippocampal neurons triggers the glutamatergic synaptogenesis, hence corroborating previous reports (Dickman et al., 2008; Eroglu et al., 2009; Kurshan et al., 2009). Moreover, we found that upregulation of the α2δ3, but not α2δ1, subunit increases the number of GABAergic synapses in hippocampal cultures already 2 weeks *in vitro*.

### α2δ3 selectively promotes axonal outgrowth and branching in inhibitory neurons

Apart from mediating the synaptic inhibition, GABA is directly involved in a variety of fundamental processes, such as neuronal migration, differentiation, and axonal outgrowth, that take place before the formation of functional synapses (Owens and Kriegstein, 2002; Huang et al., 2007). Given the α2δ3-specific effect on the GABA-dependent inhibitory postsynaptic currents (Fig. 4*F*) and the inhibitory synaptogenesis (Fig. 7*E*), next we examined whether upregulation of this subunit is associated with enhanced axonal outgrowth, as it was shown for α2δ2 subunit in the spinal cord (Tedeschi et al., 2016). Therefore, we first looked at rat hippocampal cultures, which were infected with α2δ1-HA or α2δ3-HA subunits at DIV2-DIV4 and additionally transfected at DIV4 with GFP as a volume marker to aid identification of individual neurons and their processes within the network. At DIV9-DIV10, cultures were stained for MAP2 to label the dendritic arbor of individual neurons. Subsequently, the length and branching of axons, which were detected by GFP-positive but MAP2-negative signal, were analyzed using Scholl analysis and Simple Neurite Tracer plug-in for Fiji software (Longair et al., 2011) for semiautomatic reconstruction of cells (for details, see Materials and Methods). We found no significant effect of α2δ1 or α2δ3 upregulation on the mean axonal length, nor were the number of branching points markedly affected. However, individual values obtained in α2δ3-overexpressing neurons were distributed within substantially broader range (length_min-max_ 15%-394%, mean 121 ± 20%; branches_min-max_ 32%-402%, mean 124 ± 20%; *n* = 23 neurons), compared with control (length_min-max_ 36%-208%, mean 100 ± 11%; branches_min-max_ 32%-229%, mean 100 ± 12%; *n* = 21) or α2δ1-overexpressing (length_min-max_ 15%-211%, mean 86 ± 13%; branches_min-max_ 11%-192%, mean 77 ± 11%; *n* = 21) neurons. We assumed that such heterogeneity in the dataset might reflect a mixture of values obtained in excitatory and inhibitory neurons. Therefore, we proceeded with the analysis of the axonal outgrowth and branching specifically in interneurons.

**Figure 8.**
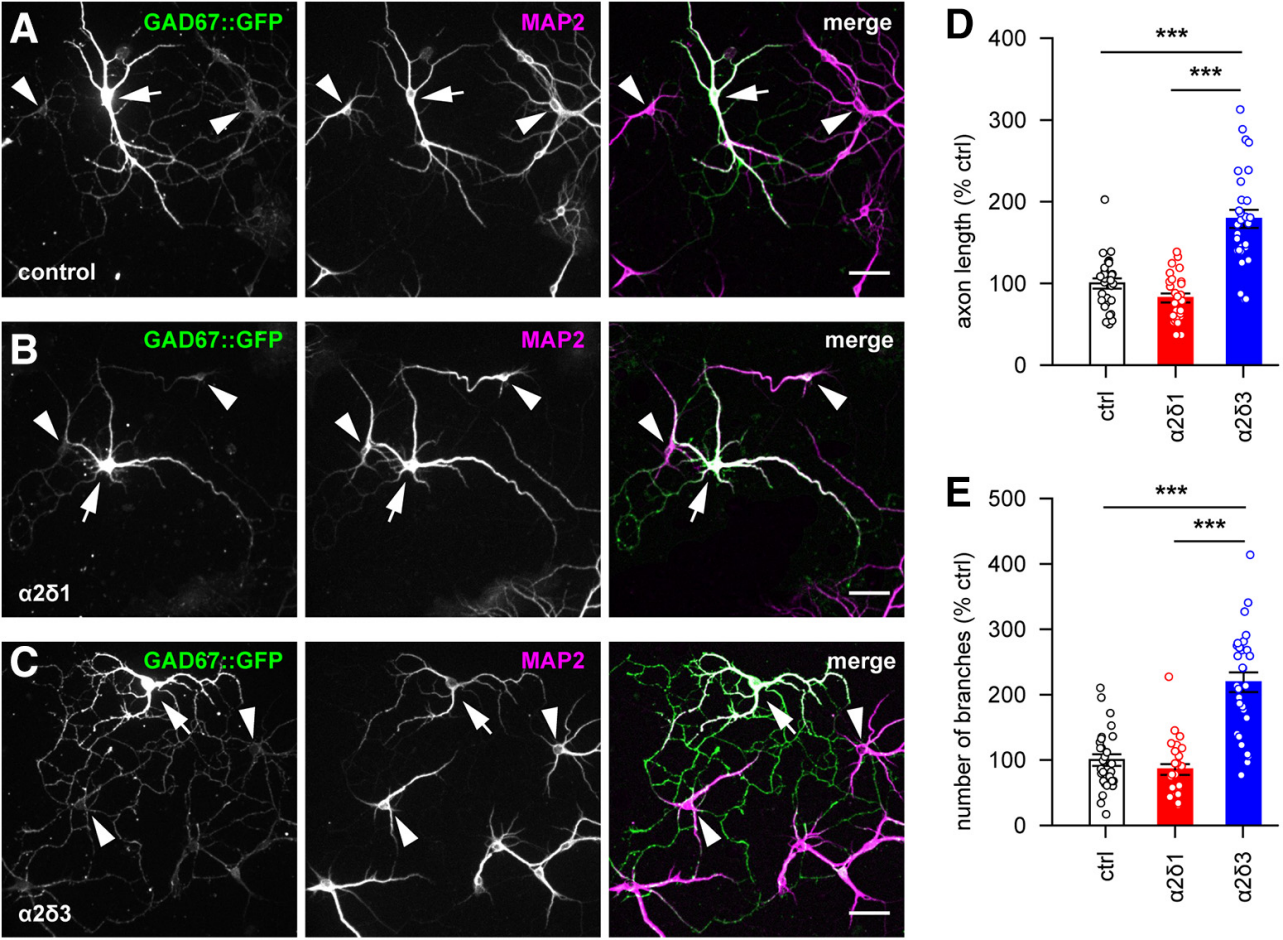
Overexpression of the α2δ3 subunit during the first week *in vitro* promotes axonal outgrowth and branching in young interneurons. ***A-C***, Representative images of GAD67::GFP mouse hippocampal neurons at DIV9 in control conditions (***A***), as well as after lentiviral infection at DIV2-DIV4 with either pLenti-syn-α2δ1::HA (***B***) or pLenti-syn-α2δ3::HA (***C***). The length and the branching of axons were analyzed exclusively in GAD67-positive interneurons (arrows), which were identified among other neurons (arrowheads) by GFP immunofluorescence. Scale bars, 50 µm. ***D***, Upregulation of the α2δ3, but not α2δ1, subunit promotes the axonal outgrowth in GAD67-positive interneurons compared with controls. ***E***, Overexpression of α2δ3 during the first developmental week leads to twofold increase in axonal branching compared with α2δ1-overexpressing or control cultures. GAD67, glutamic acid decarboxylase isoform 67, MAP2, microtubule-associated protein 2. ***p < 0.001. Means and *n* values are given in Extended Data Figure 8-1.

In young neurons, GAD67 is a rate-limiting enzyme responsible for up to 90% of GABA synthesis in the brain (Asada et al., 1997). In order to unequivocally identify and quantify individual interneurons, we prepared hippocampal cultures from mice expressing GFP under control of GAD67 promoter (GAD67::GFP). Cultures underwent the infection at DIV2-DIV4 and fixation at DIV9, followed by immunostaining for MAP2 to visualize the dendritic arbor as previously described. Subsequently, the length and the number of axonal branches were quantified exclusively for GFP-positive cells (i.e., for GAD67-positive interneurons) (Fig. 8*A-C*). In α2δ1-overexpressing interneurons, the mean axon length and the number of branches did not significantly differ from respective values obtained in control noninfected cultures. In contrast, axons of α2δ3-overexpressing interneurons were significantly longer and branched more extensively, compared with controls or α2δ1-overexpressing cultures (both *p* < 0.001 Dunn's test; Fig. 8*D*,*E*; see Extended Data Fig. 8-1). These data demonstrated that upregulation of auxiliary α2δ3 subunit of calcium channels promotes the axonal outgrowth specifically in inhibitory GABAergic interneurons.

10.1523/JNEUROSCI.1707-19.2020.f8-1 Figure 8-1 Figure 8 D. Mean axon length in GAD67-positive interneurons (normalized to mean values in control sister cultures). Figure 8 E. Mean number of axonal branches in GAD67-positive interneurons (normalized to mean values in control sister cultures). Download Figure 8-1, DOCX file

Together, our findings demonstrate that the α2δ1 or α2δ3 calcium channel subunits play an important role in several aspects of early circuitry formation in neuronal networks. The expression of both α2δ1 and α2δ3 favors the formation of synaptic connectivity. However, we found that the impact of the α2δ3 subunit is inhibitory cell type-specific, with α2δ3 upregulation being associated with enhanced GABA release, formation of inhibitory synapses, and axonal outgrowth in interneurons. Furthermore, we found that such synapse type-specific impact of α2δ1 and α2δ3 on the neurotransmitter release is associated with their functional preference for distinct VGCC isoforms.

## Discussion

This study characterizes the differential impact of α2δ1 and α2δ3 auxiliary subunits of VGCCs on structural and functional properties of developing hippocampal neurons. To overcome the limitations and side effects of constitutive KO of individual subunits of calcium channels (Striessnig and Koschak, 2008), in this work we used lentiviral overexpression of α2δ subunits in cultured neuronal networks. We found that both α2δ1 and α2δ3 can trigger excitatory synaptogenesis in hippocampal neurons, whereas upregulation of only α2δ3 subunit increases inhibitory synapse number and enhances presynaptic GABA release. Using hippocampal cultures prepared from GAD67::GFP mice, we found that α2δ3 overexpression also promotes the axon outgrowth in young interneurons. Together, these findings shed new light on the earlier reported functional redundancy of α2δ1 and α2δ3 despite pronounced structural differences between these isoforms (Klugbauer et al., 1999; Dolphin, 2013), and show their differential but complementary roles in early circuitry formation.

Throughout the experiments, we implemented two infection protocols. Lentiviral infection at different developmental time points, namely, after first, second, or third week *in vitro* (Fig. 3*D*), demonstrated that α2δ subunits alter neuronal firing and network interaction in a development-dependent and subunit-specific manner. Given the isolation of neuronal cultures from external sensory inputs that drive network activity already in the early postnatal period (Khazipov et al., 2004), the suppression of activity on α2δ3 upregulation (Fig. 3*G*) indicated a prevalence of inhibition over excitation. In contrast, α2δ1 upregulation after the second week *in vitro* consistently enhanced the network activity and demonstrated a shift toward excitation on the network level. Thus, these results show that α2δ1 and α2δ3 are intimately involved into the establishment and modulation of the excitation/inhibition balance.

To characterize the long-term consequences on neurotransmitter release, in the rest of experiments, the infection wasperformed during the first week *in vitro* and the data were acquired within the period of DIV7 to DIV24. This protocol revealed that α2δ1 overexpression selectively enhances spontaneous presynaptic glutamate release without affecting the spontaneous release of GABA (Fig. 4*C*,*F*), whereas the knockdown of this subunit led to impairment of glutamate release (Fig. 5*E*,*F*). Such selectivity of α2δ1 in facilitation of release in excitatory synapses is consistent with previously shown localization of α2δ1 primarily in excitatory presynaptic terminals in the hippocampus (Hill et al., 1993; Bian et al., 2006; Nieto-Rostro et al., 2014) and corroborates recent reports on the positive correlation between surface expression of α2δ1 and the mEPSC frequency (Cordeira et al., 2014; Zhou and Luo, 2015). Notably, higher frequency of spontaneous glutamate release in 2-week-old α2δ1-overexpressing neurons (Fig. 4*C*) was not accompanied by higher synaptic density (Fig. 7*D*), suggesting that the elevation of the release probability precedes the synaptogenic function of α2δ1.

One of the central findings of our study is the α2δ3 overexpression-induced increase in the frequency of spontaneous GABA release (Fig. 4*E*,*F*), which was accompanied by the higher density of inhibitory synapses (Fig. 7*E*). Surprisingly, we found that the α2δ3 upregulation also increases the excitatory synapse density in rather mature 3-week-old cultures (Fig. 7*D*) without affecting the mEPSC frequency (Fig. 4*B*,*C*). Electrical activity per se in immature networks is necessary and sufficient for synaptogenesis and early circuitry formation (Ben-Ari, 2001; Spitzer, 2006) and can potently influence the development of GABAergic synapses (Chattopadhyaya et al., 2007). The enhancement of the network activity observed on overexpression (Fig. 3*G*), but not downregulation (Fig. 6*P*,*Q*), after DIV7 in cultures grown on MEAs could therefore indirectly trigger the formation of surplus glutamatergic synapses.

The GABA synthesis and signaling begin already at embryonic stages; thus, GABA acts as a trophic factor influencing fundamental developmental processes before it becomes a principal inhibitory neurotransmitter (Owens and Kriegstein, 2002; Ben-Ari et al., 2007; Huang et al., 2007). Although still debated in the literature, GABA in immature neurons can exert an excitatory action so that binding to GABA_A_ receptors results in membrane depolarization. In particular, the GABA_A_ receptor-mediated depolarization in young neurons was shown to be sufficient for VGCC activation (Leinekugel et al., 1995; LoTurco et al., 1995; Ganguly et al., 2001) and required for formation and/or maintenance of GABAergic synapses (Oh et al., 2016). Intriguingly, we observed a dramatic change in the effect of α2δ3, but not α2δ1, overexpression on neuronal firing depending on the developmental stage (Fig. 3*G*). A reversal from enhancing spontaneous network activity at DIV14 to its suppression at DIV21 likely reflected the switch to hyperpolarizing GABA action and/or formation of functional inhibitory synapses (Fig. 7*E*), which requires binding of GABA to GABA_A_ receptors followed by aggregation of postsynaptic Gephyrin puncta (Oh et al., 2016). In line with increased GABA release (Fig. 4*F*), we found that the number of Bassoon puncta colocalized with Gephyrin already by the end of second developmental week was bigger in α2δ3-overexpressing cultures than in controls (Fig. 7*E*). These outcomes corroborate previous reports showing an important role of spontaneous Ca^2+^ transients in regulation of the neurite outgrowth and branching (Ciccolini et al., 2003) and structural maturation of synapses (Choi et al., 2014). Despite our data on the inhibitory synapses number (Fig. 7*E*) and GABA release (Fig. 4*E*,*F*), the finding that upregulation of α2δ3 enhances the axonal growth and branching specifically in interneurons (Fig. 8*D*,*E*) provided additional evidence for the α2δ3-specific modulation of GABA-related functions. Importantly, the outgrowth was promoted selectively in interneurons positive for GAD67, which plays major role in GABA synthesis (Asada et al., 1997), as well as in maturation of perisomatic inhibition and elimination of excessive excitatory synapses (Nakayama et al., 2012).

The α2δ subunits are known to support the trafficking of the pore-forming α1 subunit of calcium channels (Dolphin, 2012). Our results, that the elevation of frequency of neurotransmitter release in 2-week-old neurons is abolished by VGCC isoform-specific blockers (Fig. 4*I*,*J*), demonstrated a preference of α2δ1 and α2δ3 for trafficking of Cav2.1 in glutamatergic and Cav2.2 in GABAergic synapses, respectively. Agatoxin and gabapentin, but not conotoxin, were reported earlier to induce an identical nonadditive decrease in K^+^-triggered Ca^2+^ influx (Fink et al., 2000), indicating that α2δ1-mediated contribution to calcium signaling is sensitive to Cav2.1 blockade. Furthermore, our finding that α2δ1 modulates the release in excitatory synapses preferentially via P/Q-type channels is in line with strong reduction of spontaneous (Bomben et al., 2016) and evoked (Mallmann et al., 2013) release of glutamate reported in Ca_V_2.1 KO mice. Although, P/Q-type channels were shown to induce synaptic recruitment of Bassoon (Davydova et al., 2014), in our study the accumulation of Bassoon was evident only in inhibitory synapses and only on α2δ3 overexpression. Since both evoked and spontaneous GABA release require presynaptic accumulation of VGCCs (Williams et al., 2012), the latter supports previous reports (Hoppa et al., 2012; Schneider et al., 2015) and suggests that both α2δ1 and α2δ3 subunits can serve as rather universal cargos of the pore-forming α1 subunit. The α2δ isoform-specific recruitment of P/Q- or N-type channels can therefore be related to different roles α2δ subunits play in the neuronal network development. Indeed, the dominant role of the α2δ3 subunit in the early and the α2δ1 subunit in the late development matches expression profiles of P/Q- and N-type calcium channels (Scholz and Miller, 1995; Iwasaki et al., 2000; Fedchyshyn and Wang, 2005). Furthermore, the effects of the α2δ1 on the mEPSC frequency and of the α2δ3 on the mIPSC frequency were more pronounced in the presence of conotoxin and agatoxin, respectively, compared with values in respective groups obtained without toxins. The latter finding corroborates the concept of functional competition of VGCC isoforms in presynaptic active zone (Cao and Tsien, 2010; Davydova et al., 2014). In this context, the surface interaction with synaptic adhesion molecules, such as α-neurexin (Missler et al., 2003), which has been suggested in several systems (Tong et al., 2017; Brockhaus et al., 2018), can be an important contributing factor for such specificity of α2δ subunits in network development.

Our data provide support for the reported association of *CACNA2D1* and *CACNA2D3* genetic aberrations with autism (Iossifov et al., 2012; De Rubeis et al., 2014; Vergult et al., 2015) and the high comorbidity of epilepsy in individuals with autism (Tuchman and Rapin, 2002; Levisohn, 2007). By fostering the GABAergic signaling, the α2δ3 subunit effectively drives the early network activity that is crucial for the initial circuitry formation. The impact of the α2δ1 subunit becomes prominent later in development and is rather restricted to glutamatergic signaling. One interaction partner for this action could be α-neurexin, which, together with α2δ1, facilitates the trafficking of Ca_V_2.1 VGCCs to presynaptic terminals (Brockhaus et al., 2018), whereas α2δ3 may play an opposite role (Tong et al., 2017). Altered expression of α2δ1 or α2δ3 can therefore cause a chronic imbalance between excitation and inhibition that is rather characteristic for autism spectrum disorders (Rubenstein and Merzenich, 2003; Nelson and Valakh, 2015). As a consequence, impairment of α2δ-mediated functions during critical developmental periods can trigger in affected individuals devastating maladaptive changes on the network level and potentially lead to global aberrations in the brain connectivity (Baron-Cohen and Belmonte, 2005; Courchesne and Pierce, 2005) and the neural information processing (Belmonte et al., 2004).

## References

[B1] Arikkath J, Campbell KP (2003) Auxiliary subunits: essential components of the voltage-gated calcium channel complex. Curr Opin Neurobiol 13:298–307. 10.1016/s0959-4388(03)00066-7 12850214

[B2] Asada H, Kawamura Y, Maruyama K, Kume H, Ding RG, Kanbara N, Kuzume H, Sanbo M, Yagi T, Obata K (1997) Cleft palate and decreased brain gamma-aminobutyric acid in mice lacking the 67-kDa isoform of glutamic acid decarboxylase. Proc Natl Acad Sci U S A 94:6496–6499. 10.1073/pnas.94.12.6496 9177246 PMC21078

[B3] Barclay J, Balaguero N, Mione M, Ackerman SL, Letts VA, Brodbeck J, Canti C, Meir A, Page KM, Kusumi K, Perez-Reyes E, Lander ES, Frankel WN, Gardiner RM, Dolphin AC, Rees M (2001) Ducky mouse phenotype of epilepsy and ataxia is associated with mutations in the Cacna2d2 gene and decreased calcium channel current in cerebellar Purkinje cells. J Neurosci 21:6095–6104. 11487633 10.1523/JNEUROSCI.21-16-06095.2001 PMC6763162

[B4] Baron-Cohen S, Belmonte MK (2005) Autism: a window onto the development of the social and the analytic brain. Annu Rev Neurosci 28:109–126. 10.1146/annurev.neuro.27.070203.144137 16033325

[B5] Bauer CS, Nieto-Rostro M, Rahman W, Tran-Van-Minh A, Ferron L, Douglas L, Kadurin I, Sri Ranjan Y, Fernandez-Alacid L, Millar NS, Dickenson AH, Lujan R, Dolphin AC (2009) The increased trafficking of the calcium channel subunit alpha2delta-1 to presynaptic terminals in neuropathic pain is inhibited by the alpha2delta ligand pregabalin. J Neurosci 29:4076–4088. 10.1523/JNEUROSCI.0356-09.2009 19339603 PMC6665374

[B6] Belmonte MK, Cook EH Jr., Anderson GM, Rubenstein JL, Greenough WT, Beckel-Mitchener A, Courchesne E, Boulanger LM, Powell SB, Levitt PR, Perry EK, Jiang YH, DeLorey TM, Tierney E (2004) Autism as a disorder of neural information processing: directions for research and targets for therapy. Mol Psychiatry 9:646–663. 10.1038/sj.mp.4001499 15037868

[B7] Ben-Ari Y (2001) Developing networks play a similar melody. Trends in neurosciences 24:353–360. 10.1016/s0166-2236(00)01813-0 11356508

[B8] Ben-Ari Y, Gaiarsa JL, Tyzio R, Khazipov R (2007) GABA: a pioneer transmitter that excites immature neurons and generates primitive oscillations. Physiol Rev 87:1215–1284. 10.1152/physrev.00017.2006 17928584

[B9] Bettencourt LM, Stephens GJ, Ham MI, Gross GW (2007) Functional structure of cortical neuronal networks grown in vitro. Physical review E, Statistical, nonlinear, and soft matter physics 75:021915. 10.1103/PhysRevE.75.021915 17358375

[B10] Bian F, Li Z, Offord J, Davis MD, McCormick J, Taylor CP, Walker LC, (2006) Calcium channel alpha2-delta type 1 subunit is the major binding protein for pregabalin in neocortex, hippocampus, amygdala, and spinal cord: an ex vivo autoradiographic study in alpha2-delta type 1 genetically modified mice. Brain Res 1075:68–80. 10.1016/j.brainres.2005.12.084 16460711

[B11] Bikbaev A, Frischknecht R, Heine M (2015) Brain extracellular matrix retains connectivity in neuronal networks. Sci Rep 5:14527. 10.1038/srep14527 26417723 PMC4586818

[B12] Bomben VC, Aiba I, Qian J, Mark MD, Herlitze S, Noebels JL (2016) Isolated P/Q Calcium Channel Deletion in Layer VI Corticothalamic Neurons Generates Absence Epilepsy. J Neurosci 36:405–418. 10.1523/JNEUROSCI.2555-15.2016 26758833 PMC4710767

[B13] Brockhaus J, Schreitmuller M, Repetto D, Klatt O, Reissner C, Elmslie K, Heine M, Missler M (2018) alpha-Neurexins Together with alpha2delta-1 Auxiliary Subunits Regulate Ca(2+) Influx through Cav2.1 Channels. J Neurosci 38:8277–8294. 10.1523/JNEUROSCI.0511-18.2018 30104341 PMC6596161

[B14] Cao YQ, Tsien RW (2010) Different relationship of N- and P/Q-type Ca2+ channels to channel-interacting slots in controlling neurotransmission at cultured hippocampal synapses. J Neurosci 30:4536–4546. 10.1523/JNEUROSCI.5161-09.2010 20357104 PMC3842455

[B15] Catterall WA (2000) From ionic currents to molecular mechanisms: the structure and function of voltage-gated sodium channels. Neuron 26:13–25. 10.1016/s0896-6273(00)81133-2 10798388

[B16] Chattopadhyaya B, Di Cristo G, Wu CZ, Knott G, Kuhlman S, Fu Y, Palmiter RD, Huang ZJ (2007) GAD67-mediated GABA synthesis and signaling regulate inhibitory synaptic innervation in the visual cortex. Neuron 54:889–903. 10.1016/j.neuron.2007.05.015 17582330 PMC2077924

[B17] Choi BJ, Imlach WL, Jiao W, Wolfram V, Wu Y, Grbic M, Cela C, Baines RA, Nitabach MN, McCabe BD (2014) Miniature neurotransmission regulates Drosophila synaptic structural maturation. Neuron 82:618–634. 10.1016/j.neuron.2014.03.012 24811381 PMC4022839

[B18] Ciccolini F, Collins TJ, Sudhoelter J, Lipp P, Berridge MJ, Bootman MD (2003) Local and global spontaneous calcium events regulate neurite outgrowth and onset of GABAergic phenotype during neural precursor differentiation. J Neurosci 23:103–111. 12514206 10.1523/JNEUROSCI.23-01-00103.2003 PMC6742163

[B19] Cole RL, Lechner SM, Williams ME, Prodanovich P, Bleicher L, Varney MA, Gu G (2005) Differential distribution of voltage-gated calcium channel alpha-2 delta (alpha2delta) subunit mRNA-containing cells in the rat central nervous system and the dorsal root ganglia. J Comp Neur 491:246–269. 10.1002/cne.20693 16134135

[B20] Cordeira JW, Felsted JA, Teillon S, Daftary S, Panessiti M, Wirth J, Sena-Esteves M, Rios M (2014) Hypothalamic dysfunction of the thrombospondin receptor alpha2delta-1 underlies the overeating and obesitytriggered by brain-derived neurotrophic factor deficiency. J Neurosci 34:554–565. 10.1523/JNEUROSCI.1572-13.2014 24403154 PMC3870936

[B21] Courchesne E, Pierce K (2005) Brain overgrowth in autism during a critical time in development: implications for frontal pyramidal neuron and interneuron development and connectivity. Int J Dev Neurosci 23:153–170. 10.1016/j.ijdevneu.2005.01.003 15749242

[B22] Davydova D, Marini C, King C, Klueva J, Bischof F, Romorini S, Montenegro-Venegas C, Heine M, Schneider R, Schroder MS, Altrock WD, Henneberger C, Rusakov DA, Gundelfinger ED, Fejtova A (2014) Bassoon specifically controls presynaptic P/Q-type Ca(2+) channels via RIM-binding protein. Neuron 82:181–194. 10.1016/j.neuron.2014.02.012 24698275

[B23] De Rubeis S, He X, Goldberg AP, Poultney CS, Samocha K, Ercument Cicek A, Kou Y, Liu L, Fromer M, Walker S, Singh T, Klei L, Kosmicki J, Fu S-C, Aleksic B, Biscaldi M, Bolton PF, Brownfeld JM, Cai J, The DDD Study et al (2014) Synaptic, transcriptional and chromatin genes disrupted in autism. Nature 515:209–215. 10.1038/nature13772 25363760 PMC4402723

[B24] Dickman DK, Kurshan PT, Schwarz TL (2008) Mutations in a Drosophila alpha2delta voltage-gated calcium channel subunit reveal a crucial synaptic function. J Neurosci 28:31–38. 10.1523/JNEUROSCI.4498-07.2008 18171920 PMC6671140

[B25] Dolphin AC (2012) Calcium channel auxiliary alpha2delta and beta subunits: trafficking and one step beyond. Nature reviews Neuroscience 13:542–555. 10.1038/nrn3311 22805911

[B26] Dolphin AC (2013) The alpha2delta subunits of voltage-gated calcium channels. Biochim Biophys Acta 1828:1541–1549. 10.1016/j.bbamem.2012.11.019 23196350

[B27] Edvardson S, Oz S, Abulhijaa FA, Taher FB, Shaag A, Zenvirt S, Dascal N, Elpeleg O (2013) Early infantile epileptic encephalopathy associated with a high voltage gated calcium channelopathy. J Med Genet 50:118–123. 10.1136/jmedgenet-2012-101223 23339110

[B28] Ermolyuk YS, Alder FG, Surges R, Pavlov IY, Timofeeva Y, Kullmann DM, Volynski KE (2013) Differential triggering of spontaneous glutamate release by P/Q-, N- and R-type Ca2+ channels. Nat Neurosci 16:1754–1763. 10.1038/nn.3563 24185424 PMC4176737

[B29] Eroglu C, Allen NJ, Susman MW, O'Rourke NA, Park CY, Ozkan E, Chakraborty C, Mulinyawe SB, Annis DS, Huberman AD, Green EM, Lawler J, Dolmetsch R, Garcia KC, Smith SJ, Luo ZD, Rosenthal A, Mosher DF, Barres BA (2009) Gabapentin receptor alpha2delta-1 is a neuronal thrombospondin receptor responsible for excitatory CNS synaptogenesis. Cell 139:380–392. 10.1016/j.cell.2009.09.025 19818485 PMC2791798

[B30] Fedchyshyn MJ, Wang LY (2005) Developmental transformation of the release modality at the calyx of Held synapse. J Neurosci 25:4131–4140. 10.1523/JNEUROSCI.0350-05.2005 15843616 PMC6724960

[B31] Felsted JA, Chien CH, Wang D, Panessiti M, Ameroso D, Greenberg A, Feng G, Kong D, Rios M (2017) Alpha2delta-1 in SF1(+) Neurons of the Ventromedial Hypothalamus Is an Essential Regulator of Glucose and Lipid Homeostasis. Cell Rep 21:2737–2747. 10.1016/j.celrep.2017.11.048 29212022 PMC5730076

[B32] Fink K, Meder W, Dooley DJ, Gothert M (2000) Inhibition of neuronal Ca(2+) influx by gabapentin and subsequent reduction of neurotransmitter release from rat neocortical slices. Br J Pharmacol 130:900–906. 10.1038/sj.bjp.0703380 10864898 PMC1572136

[B33] Folstein SE, Rosen-Sheidley B (2001) Genetics of autism: complex aetiology for a heterogeneous disorder. Nat Rev Genet 2:943–955. 10.1038/35103559 11733747

[B34] Freitag CM (2007) The genetics of autistic disorders and its clinical relevance: a review of the literature. Mol Psychiatry 12:2–22. 10.1038/sj.mp.4001896 17033636

[B35] Fuller-Bicer GA, Varadi G, Koch SE, Ishii M, Bodi I, Kadeer N, Muth JN, Mikala G, Petrashevskaya NN, Jordan MA, Zhang S-P, Qin N, Flores CM, Isaacsohn I, Varadi M, Mori Y, Jones WK, Schwartz A (2009) Targeted disruption of the voltage-dependent calcium channel alpha2/delta-1-subunit. Am J Physiol Heart Circ Physiol 297:H117–H124. 19429829 10.1152/ajpheart.00122.2009 PMC2711723

[B36] Ganguly K, Schinder AF, Wong ST, Poo M (2001) GABA itself promotes the developmental switch of neuronal GABAergic responses from excitation to inhibition. Cell 105:521–532. 10.1016/s0092-8674(01)00341-5 11371348

[B37] Geisler S, Schopf CL, Obermair GJ (2015) Emerging evidence for specific neuronal functions of auxiliary calcium channel alpha(2)delta subunits. Gen Physiol Biophys 34:105–118. 10.4149/gpb_2014037 25504062 PMC4487825

[B38] Goswami SP, Bucurenciu I, Jonas P (2012) Miniature IPSCs in hippocampal granule cells are triggered by voltage-gated Ca2+ channels via microdomain coupling. J Neurosci 32:14294–14304. 10.1523/JNEUROSCI.6104-11.2012 23055500 PMC3632771

[B39] Hill DR, Suman-Chauhan N, Woodruff GN (1993) Localization of [3H]gabapentin to a novel site in rat brain: autoradiographic studies. Eur J Pharmacol 244:303–309. 10.1016/0922-4106(93)90156-4 8384571

[B40] Hoppa MB, Lana B, Margas W, Dolphin AC, Ryan TA (2012) alpha2delta expression sets presynaptic calcium channel abundance and release probability. Nature 486:122–125. 10.1038/nature11033 22678293 PMC3376018

[B41] Huang ZJ, Di Cristo G, Ango F (2007) Development of GABA innervation in the cerebral and cerebellar cortices. Nature reviews Neuroscience 8:673–686. 10.1038/nrn2188 17704810

[B42] Iossifov I, Ronemus M, Levy D, Wang Z, Hakker I, Rosenbaum J, Yamrom B, Lee Y-H, Narzisi G, Leotta A, Kendall J, Grabowska E, Ma B, Marks S, Rodgers L, Stepansky A, Troge J, Andrews P, Bekritsky M, Pradhan K, et al (2012) De novo gene disruptions in children on the autistic spectrum. Neuron 74:285–299. 10.1016/j.neuron.2012.04.009 22542183 PMC3619976

[B43] Iwasaki S, Momiyama A, Uchitel OD, Takahashi T (2000) Developmental changes in calcium channel types mediating central synaptic transmission. J Neurosci 20:59–65. 10627581 10.1523/JNEUROSCI.20-01-00059.2000 PMC6774098

[B44] Kaech S, Banker G (2006) Culturing hippocampal neurons. Nat Protoc 1:2406–2415. 10.1038/nprot.2006.356 17406484

[B45] Khazipov R, Sirota A, Leinekugel X, Holmes GL, Ben-Ari Y, Buzsáki G, (2004) Early motor activity drives spindle bursts in the developing somatosensory cortex. Nature 432:758–761. 10.1038/nature03132 15592414

[B46] Klugbauer N, Lacinova L, Marais E, Hobom M, Hofmann F (1999) Molecular diversity of the calcium channel alpha2delta subunit. J Neurosci 19:684–691. 10.1523/JNEUROSCI.19-02-00684.1999 9880589 PMC6782206

[B47] Kurshan PT, Oztan A, Schwarz TL (2009) Presynaptic alpha2delta-3 is required for synaptic morphogenesis independent of its Ca2+-channel functions. Nat Neurosci 12:1415–1423. 10.1038/nn.2417 19820706 PMC2996863

[B48] Leinekugel X, Tseeb V, Ben-Ari Y, Bregestovski P (1995) Synaptic GABAA activation induces Ca2+ rise in pyramidal cells and interneurons from rat neonatal hippocampal slices. J Physiol 487: 319–329. 10.1113/jphysiol.1995.sp020882 8558466 PMC1156575

[B49] Levisohn PM (2007) The autism-epilepsy connection. Epilepsia 48 Suppl 9:33–35. 10.1111/j.1528-1167.2007.01399.x 18047599

[B50] Longair MH, Baker DA, Armstrong JD (2011) Simple Neurite Tracer: open source software for reconstruction, visualization and analysis of neuronal processes. Bioinformatics 27:2453–2454. 10.1093/bioinformatics/btr390 21727141

[B51] LoTurco JJ, Owens DF, Heath MJ, Davis MB, Kriegstein AR (1995) GABA and glutamate depolarize cortical progenitor cells and inhibit DNA synthesis. Neuron 15:1287–1298. 10.1016/0896-6273(95)90008-x 8845153

[B52] Luo ZD, Chaplan SR, Higuera ES, Sorkin LS, Stauderman KA, Williams ME, Yaksh TL (2001) Upregulation of dorsal root ganglion (alpha)2(delta) calcium channel subunit and its correlation with allodynia in spinal nerve-injured rats. J Neurosci 21:1868–1875. 11245671 10.1523/JNEUROSCI.21-06-01868.2001 PMC6762626

[B53] Mallmann RT, Elgueta C, Sleman F, Castonguay J, Wilmes T, van den Maagdenberg A, Klugbauer N (2013) Ablation of Ca(V)2.1 voltage-gated Ca(2)(+) channels in mouse forebrain generates multiple cognitive impairments. PLoS One 8:e78598. 10.1371/journal.pone.0078598 24205277 PMC3814415

[B54] Mastrolia V, Flucher SM, Obermair GJ, Drach M, Hofer H, Renström E, Schwartz A, Striessnig J, Flucher BE, Tuluc P (2017) Loss of α(2)δ-1 Calcium Channel Subunit Function Increases the Susceptibility for Diabetes. Diabetes 66:897–907. 28115397 10.2337/db16-0336 PMC7360433

[B55] Missler M, Zhang W, Rohlmann A, Kattenstroth G, Hammer RE, Gottmann K, Sudhof TC (2003) Alpha-neurexins couple Ca2+ channels to synaptic vesicle exocytosis. Nature 423:939–948. 10.1038/nature01755 12827191

[B56] Nakayama H, Miyazaki T, Kitamura K, Hashimoto K, Yanagawa Y, Obata K, Sakimura K, Watanabe M, Kano M (2012) GABAergic inhibition regulates developmental synapse elimination in the cerebellum. Neuron 74:384–396. 10.1016/j.neuron.2012.02.032 22542190

[B57] Nelson SB, Valakh V (2015) Excitatory/Inhibitory Balance and Circuit Homeostasis in Autism Spectrum Disorders. Neuron 87:684–698. 10.1016/j.neuron.2015.07.033 26291155 PMC4567857

[B58] Nieto-Rostro M, Sandhu G, Bauer CS, Jiruska P, Jefferys JG, Dolphin AC (2014) Altered expression of the voltage-gated calcium channel subunit alpha(2)delta-1: a comparison between two experimental models ofepilepsy and a sensory nerve ligation model of neuropathic pain. Neuroscience 283:124–137. 10.1016/j.neuroscience.2014.03.013 24641886 PMC4259901

[B59] Obermair GJ, Kugler G, Baumgartner S, Tuluc P, Grabner M, Flucher BE (2005) The Ca2+ channel alpha2delta-1 subunit determines Ca2+ current kinetics in skeletal muscle but not targeting of alpha1S or excitation-contraction coupling. J Biol Chem 280:2229–2237. 15536090 10.1074/jbc.M411501200

[B60] Obermair GJ, Schlick B, Di Biase V, Subramanyam P, Gebhart M, Baumgartner S, Flucher BE (2010) Reciprocal interactions regulate targeting of calcium channel beta subunits and membrane expression of alpha1 subunits in cultured hippocampal neurons. J Biol Chem 285:5776–5791. 19996312 10.1074/jbc.M109.044271 PMC2820804

[B61] Oh WC, Lutzu S, Castillo PE, Kwon HB (2016) De novo synaptogenesis induced by GABA in the developing mouse cortex. Science 353:1037–1040. 10.1126/science.aaf5206 27516412 PMC5104171

[B62] Owens DF, Kriegstein AR (2002) Is there more to GABA than synaptic inhibition? Nature reviews Neuroscience 3:715–727. 10.1038/nrn919 12209120

[B63] Patel R, Bauer CS, Nieto-Rostro M, Margas W, Ferron L, Chaggar K, Crews K, Ramirez JD, Bennett DL, Schwartz A, Dickenson AH, Dolphin AC (2013) alpha2delta-1 gene deletion affects somatosensory neuron function and delays mechanical hypersensitivity in response to peripheral nerve damage. J Neurosci 33:16412–16426. 10.1523/JNEUROSCI.1026-13.2013 24133248 PMC3797367

[B64] Pippucci T, Parmeggiani A, Palombo F, Maresca A, Angius A, Crisponi L, Cucca F, Liguori R, Valentino ML, Seri M, Carelli V (2013) A novel null homozygous mutation confirms CACNA2D2 as a gene mutated in epileptic encephalopathy. PLoS One 8:e82154. 10.1371/journal.pone.0082154 24358150 PMC3864908

[B65] Risher WC, Kim N, Koh S, Choi J-E, Mitev P, Spence EF, Pilaz L-J, Wang D, Feng G, Silver DL, Soderling SH, Yin HH, Eroglu C (2018) Thrombospondin receptor α2δ-1 promotes synaptogenesis and spinogenesis via postsynaptic Rac1. J Cell Biol 217:3747–3765. 30054448 10.1083/jcb.201802057 PMC6168259

[B66] Rubenstein JL, Merzenich MM (2003) Model of autism: increased ratio of excitation/inhibition in key neural systems. Genes Brain Behav 2:255–267. 10.1034/j.1601-183x.2003.00037.x 14606691 PMC6748642

[B67] Schlick B, Flucher BE, Obermair GJ (2010) Voltage-activated calcium channel expression profiles in mouse brain and cultured hippocampal neurons. Neuroscience 167:786–798. 10.1016/j.neuroscience.2010.02.037 20188150 PMC3315124

[B68] Schneider R, Hosy E, Kohl J, Klueva J, Choquet D, Thomas U, Voigt A, Heine M (2015) Mobility of calcium channels in the presynaptic membrane. Neuron 86:672–679. 10.1016/j.neuron.2015.03.050 25892305

[B69] Scholz KP, Miller RJ (1995) Developmental changes in presynaptic calcium channels coupled to glutamate release in cultured rat hippocampal neurons. J Neurosci 15:4612–4617. 7790927 10.1523/JNEUROSCI.15-06-04612.1995 PMC6577697

[B70] Spitzer NC (2006) Electrical activity in early neuronal development. Nature 444:707–712. 10.1038/nature05300 17151658

[B71] Subramanyam P, Obermair GJ, Baumgartner S, Gebhart M, Striessnig J, Kaufmann WA, Geley S, Flucher BE (2009) Activity and calcium regulate nuclear targeting of the calcium channel beta4b subunit in nerve and muscle cells. Channels (Austin) 3:343–355. 19755859 10.4161/chan.3.5.9696 PMC2853709

[B72] Striessnig J, Koschak A (2008) Exploring the function and pharmacotherapeutic potential of voltage-gated Ca2+ channels with gene knockout models. Channels (Austin) 2:233–251. 10.4161/chan.2.4.5847 18719397

[B73] Tedeschi A, Dupraz S, Laskowski CJ, Xue J, Ulas T, Beyer M, Schultze JL, Bradke F (2016) The Calcium Channel Subunit Alpha2delta2 Suppresses Axon Regeneration in the Adult CNS. Neuron 92:419–434. 10.1016/j.neuron.2016.09.026 27720483

[B74] Tong XJ, Lopez-Soto EJ, Li L, Liu H, Nedelcu D, Lipscombe D, Hu Z, Kaplan JM (2017) Retrograde Synaptic Inhibition Is Mediated by alpha-Neurexin Binding to the alpha2delta Subunits of N-Type Calcium Channels. Neuron 95:326–340.e5. 10.1016/j.neuron.2017.06.018 28669545 PMC5548138

[B75] Tuchman R, Rapin I (2002) Epilepsy in autism. Lancet Neurol 1:352–358. 10.1016/s1474-4422(02)00160-6 12849396

[B76] van Pelt J, Wolters PS, Corner MA, Rutten WL, Ramakers GJ (2004) Long-term characterization of firing dynamics of spontaneous bursts in cultured neural networks. IEEE transactions on bio-medical engineering 51:2051–2062. 10.1109/TBME.2004.827936 15536907

[B77] Vergult S, Dheedene A, Meurs A, Faes F, Isidor B, Janssens S, Gautier A, Le Caignec C, Menten B (2015) Genomic aberrations of the CACNA2D1 gene in three patients with epilepsy and intellectual disability. Eur J Hum Genet 23:628–632. 10.1038/ejhg.2014.141 25074461 PMC4402620

[B78] Wheeler DB, Randall A, Tsien RW (1994) Roles of N-type and Q-type Ca2+ channels in supporting hippocampal synaptic transmission. Science 264:107–111. 10.1126/science.7832825 7832825

[B79] Williams C, Chen W, Lee CH, Yaeger D, Vyleta NP, Smith SM (2012) Coactivation of multiple tightly coupled calcium channels triggers spontaneous release of GABA. Nat Neurosci 15:1195–1197. 10.1038/nn.3162 22842148 PMC3431448

[B80] Zamponi GW, Striessnig J, Koschak A, Dolphin AC (2015) The Physiology, Pathology, and Pharmacology of Voltage-Gated Calcium Channels and Their Future Therapeutic Potential. Pharmacol Rev 67:821–870. 10.1124/pr.114.009654 26362469 PMC4630564

[B81] Zhou C, Luo ZD (2015) Nerve injury-induced calcium channel alpha-2-delta-1 protein dysregulation leads to increased pre-synaptic excitatory input into deep dorsal horn neurons and neuropathic allodynia. Eur J Pain 19:1267–1276. 10.1002/ejp.656 25691360 PMC4539283

