# Supplementary material for: Auxiliary α2δ1 and α2δ3 Subunits of Calcium Channels Drive Excitatory and Inhibitory Neuronal Network Development
Source: J Neurosci. 2020 Jun 17;40(25):4824–41. doi: 10.1523/JNEUROSCI.1707-19.2020 (PMC7326358; doi:10.1523/JNEUROSCI.1707-19.2020)
Supplement: Figure 1-1 [file ns-JN-RM-1707-19-s01.docx]

**Extended Figures**

**Figure 1-1.**Figure 1 E. The mean number of asymmetric synapses in the CA1 area of the hippocampus (data were normalized to the mean value (taken as 100%) obtained from wild type mice).

| group | mean±S.E.M., % | n, images | Kolmogorov-Smirnov test,  P value |
| --- | --- | --- | --- |
| wild type | 100±5 | 9 | P<.001 |
| α2δ1^-/-^ | 68±2 | 9 |  |

Figure 1 F. Presynaptic protein composition in α2δ1^-/-^ mice (data were normalized to respective mean values (taken as 100%) in wild type animals).

| protein | group | mean±S.E.M., % | n, animals | t test,  t and P values |
| --- | --- | --- | --- | --- |
| Ca_V_2.1 | wild type | 100±10 | 4 | n.s. |
|  | α2δ1^-/-^ | 96±10 | 4 |  |
| vGlut1 | wild type | 100±31 | 4 | n.s. |
|  | α2δ1^-/-^ | 76±21 | 4 |  |
| GAD65 | wild type | 100±13 | 4 | n.s. |
|  | α2δ1^-/-^ | 109±11 | 4 |  |
| Synapsin1a/1b | wild type | 100±22 | 4 | n.s. |
|  | α2δ1^-/-^ | 126±18 | 4 |  |
| Synaptophysin | wild type | 100±17 | 4 | n.s. |
|  | α2δ1^-/-^ | 85±11 | 4 |  |
| Synaptotagmin1 | wild type | 100±14 | 4 | n.s. |
|  | α2δ1^-/-^ | 126±15 | 4 |  |
| SNAP-25 | wild type | 100±32 | 3 | t=2.62, P<.05 |
|  | α2δ1^-/-^ | 28±6 | 4 |  |
| CASK | wild type | 100±16 | 4 | n.s. |
|  | α2δ1^-/-^ | 91±32 | 4 |  |
| Liprin-α3 | wild type | 100±16 | 4 | n.s. |
|  | α2δ1^-/-^ | 158±34 | 3 |  |
| Rab3A | wild type | 100±29 | 4 | n.s. |
|  | α2δ1^-/-^ | 105±12 | 4 |  |
| tyrosine hydroxylase | wild type | 100±7 | 4 | n.s. |
|  | α2δ1^-/-^ | 91±9 | 4 |  |

**Figure 2-1.**

Figure 2 G. Total protein amount of the α2δ1 subunit in neurons (data were normalized to values in non-infected sister controls taken as 100%).

| group | mean±S.E.M., % | n, cultures | t-test,  t and P values |
| --- | --- | --- | --- |
| control | 100±0 | 3 | t=3.69, P<.05 |
| α2δ1-HA | 136±10 | 3 |  |

Figure 2 H. Total protein amount of the α2δ3 subunit in neurons (data were normalized to values in non-infected sister controls taken as 100%).

| group | mean±S.E.M., % | n, cultures | t-test,  t and P values |
| --- | --- | --- | --- |
| control | 100±0 | 3 | t=4.79, P<.01 |
| α2δ3-HA | 259±33 | 3 |  |

**Figure 3-1.**

Figure 3 G. Mean spontaneous firing rate in hippocampal cultures grown on MEAs (normalized for each culture to the mean value in control group at respective DIV).

| DIV | group | mean±S.E.M., % | n, MEAs | n, channels | one-way ANOVA,  F and P values | Duncan’s test,  P value |
| --- | --- | --- | --- | --- | --- | --- |
| 14 | control | 100±19 | 6 | 137 | F_3,249_=19.8  P<.001 |  |
|  | α2δ1 | 120±24 | 5 | 124 |  | n.s. vs. control  P<.001 vs. α2δ3 |
|  | α2δ3 | 370±55 | 5 | 91 |  | P<.001 vs. control  P<.001 vs. α2δ1 |
| 21 | control | 100±10 | 8 | 281 | F_2,725_=21.1  P<.001 |  |
|  | α2δ1 | 174±14 | 8 | 357 |  | P<.01 vs. control  P<.001 vs. α2δ3 |
|  | α2δ3 | 21±4 | 8 | 90 |  | P<.001 vs. control  P<.001 vs. α2δ1 |
| 28 | control | 100±11 | 10 | 342 | F_2,795_=19.4  P<.001 |  |
|  | α2δ1 | 193±25 | 7 | 204 |  | P<.001 vs. control  P<.001 vs. α2δ3 |
|  | α2δ3 | 60±6 | 6 | 252 |  | n.s. vs. control  P<.001 vs. α2δ1 |

Figure 3 H. Mean network burst (NB) rate per min in hippocampal cultures overexpressing the α2δ subunits (normalized for each culture to the mean value in control group at respective DIV).

| DIV | group | mean±S.E.M., % | n, MEAs | n, minutes | one-way ANOVA,  F and P values | Duncan’s test,  P value |
| --- | --- | --- | --- | --- | --- | --- |
| 14 | control | 100±14 | 6 | 60 | F_2,157_=2.9  P=.054 |  |
|  | α2δ1 | 112±24 | 5 | 50 |  |  |
|  | α2δ3 | 200±50 | 5 | 50 |  |  |
| 21 | control | 100±5 | 8 | 80 | F_2,237_=279.1  P<.001 |  |
|  | α2δ1 | 205±8 | 8 | 80 |  | P<.001 vs. control  P<.001 vs. α2δ3 |
|  | α2δ3 | 18±2 | 8 | 80 |  | P<.001 vs. control  P<.001 vs. α2δ1 |
| 28 | control | 100±5 | 10 | 100 | n.s. |  |
|  | α2δ1 | 96±11 | 7 | 70 |  |  |
|  | α2δ3 | 92±5 | 6 | 60 |  |  |

Figure 3 I. Mean burst onset lag in hippocampal cultures overexpressing the α2δ subunits.

| DIV | group | mean±S.E.M., ms | n, MEAs | n, minutes | one-way ANOVA,  F and P values | Duncan’s test,  P value |
| --- | --- | --- | --- | --- | --- | --- |
| 14 | control | 66±5 | 6 | 53 | F_2,105_=8.4  P<.001 |  |
|  | α2δ1 | 42±7 | 5 | 30 |  | P<.01 vs. control  n.s. vs. α2δ3 |
|  | α2δ3 | 38±4 | 5 | 25 |  | P<.01 vs. control  n.s. vs. α2δ1 |
| 21 | control | 53±3 | 8 | 80 | F_2,197_=28.8  P<.001 |  |
|  | α2δ1 | 23±1 | 8 | 80 |  | P<.001 vs. control  P<.001 vs. α2δ3 |
|  | α2δ3 | 72±11 | 8 | 40 |  | P<.01 vs. control  P<.001 vs. α2δ1 |
| 28 | control | 30±2 | 10 | 100 | F_2,220_=3.1  P<.05 |  |
|  | α2δ1 | 25±2 | 7 | 63 |  | n.s. vs. control  P<.05 vs. α2δ3 |
|  | α2δ3 | 32±3 | 6 | 60 |  | n.s. vs. control  P<.05 vs. α2δ1 |

**Figure 4-1.**

Figure 4 C. Mean mEPSC frequency (normalized to control mean at respective DIV).

| DIV | group | frequency ± S.E.M. (Hz) | | n, neurons | K-W ANOVA,  H and P values | Dunn’s test,  P value |
| --- | --- | --- | --- | --- | --- | --- |
| 7-11 | control | 0.68 ± 0.05 | 15 | | n.s. |  |
|  | α2δ1 | 0.69 ± 0.06 | 15 | |  |  |
|  | α2δ3 | 0.83 ± 0.08 | 14 | |  |  |
| 14-17 | control | 2.11 ± 0.65 | 9 | | H_3,37_=14.2  P<.001 |  |
|  | α2δ1 | 5.0 ± 0.57 | 15 | |  | P<.01 vs. control  P<.05 vs. α2δ3 |
|  | α2δ3 | 2.67 ± 0.44 | 13 | |  | n.s. vs. control  P<.05 vs. α2δ1 |
| 18-24 | control | 0.73 ± 0.13 | 12 | |  |  |
|  | α2δ1 | 1.39 ± 0.27 | 12 | | H_3,34_=8.2  P<.05 | P<.05 vs. control  P<.05 vs. α2δ3 |
|  | α2δ3 | 0.82 ± 0.28 | 10 | |  | n.s. vs. control  n.s. vs. α2δ1 |

Figure 4 D. Mean mEPSC amplitude (normalized to control mean at respective DIV).

| DIV | group | mean amplitude ± S.E.M. (pA) | n, neurons | K-W ANOVA,  H and P values | Dunn’s test,  P value |
| --- | --- | --- | --- | --- | --- |
| 7-11 | control | 16.1 ± 0.9 | 15 | n.s. |  |
|  | α2δ1 | 16.6 ± 1.1 | 15 |  |  |
|  | α2δ3 | 18.1 ± 2.5 | 14 |  |  |
| 14-17 | control | 26.3 ± 3.4 | 9 | n.s. |  |
|  | α2δ1 | 24.2 ± 2.8 | 15 |  |  |
|  | α2δ3 | 26.6 ± 2.0 | 13 |  |  |
| 18-24 | control | 15.9 ± 2.9 | 12 | n.s. |  |
|  | α2δ1 | 18.1 ± 1.1 | 12 |  |  |
|  | α2δ3 | 16.3 ± 2.0 | 10 |  |  |

Figure 4 F. Mean mIPSC frequency (normalized to control mean at respective DIV).

| DIV | group | frequency ± S.E.M. (Hz) | n, neurons | K-W ANOVA,  H and P values | Dunn’s test,  P value |
| --- | --- | --- | --- | --- | --- |
| 7-11 | control | 1.09 ± 0.09 | 15 |  |  |
|  | α2δ1 | 1.38 ± 0.24 | 14 | n.s. |  |
|  | α2δ3 | 1.7 ± 0.25 | 16 |  |  |
| 14-17 | control | 1.72 ± 0.26 | 10 | H_3,31_=18.5  P<.001 |  |
|  | α2δ1 | 2.59 ± 0.38 | 13 |  | n.s. vs. control  n.s. vs. α2δ3 |
|  | α2δ3 | 7.34 ± 1.15 | 8 |  | P<.001 vs. control  P<.05 vs. α2δ1 |
| 18-24 | control | 0.55 ± 0.09 | 10 | H_3,36_=6.7  P<.05 |  |
|  | α2δ1 | 0.9 ± 0.2 | 12 |  | n.s. vs. control  n.s. vs. α2δ3 |
|  | α2δ3 | 1.05 ± 0.17 | 14 |  | P<.05 vs. control  n.s. vs. α2δ1 |

Figure 4 G. Mean mIPSC amplitude (normalized to control mean at respective DIV).

| DIV | group | mean amplitude ± S.E.M. (pA) | n, neurons | K-W ANOVA,  H and P values | Dunn’s test,  P value |
| --- | --- | --- | --- | --- | --- |
| 7-11 | control | 51.7 ± 9.8 | 15 | n.s. |  |
|  | α2δ1 | 52.4 ± 5.0 | 14 |  |  |
|  | α2δ3 | 54.3 ± 4.8 | 16 |  |  |
| 14-17 | control | 51.7 ± 4.9 | 10 | n.s. |  |
|  | α2δ1 | 55.4 ± 5.8 | 13 |  |  |
|  | α2δ3 | 49.1 ± 4.0 | 8 |  |  |
| 18-24 | control | 57.1 ± 11.3 | 10 | H_3,36_=9.3  P<.01 |  |
|  | α2δ1 | 65.8 ± 3.5 | 12 |  | n.s. vs. control  P<.01 vs. α2δ3 |
|  | α2δ3 | 50.6 ± 3.8 | 14 |  | n.s. vs. control  P<.01 vs. α2δ1 |

Figure 4 H. Mean mEPSC and mIPSC frequency (normalized to corresponding mean value in respective control groups).

| current | group | mean±S.E.M., % | n, neurons | Mann-Whitney test,  p value |
| --- | --- | --- | --- | --- |
| mEPSC | control | 100±31 | 9 |  |
|  | α2δ1 | 230±36 | 9 | P<.01 vs. control |
|  | α2δ3 | 121±25 | 11 | n.s. vs. control |
|  | Cd^2+^ control | 38±9 | 7 | P<.01 vs. control |
|  | Cd^2+^ α2δ1 | 31±3 | 6 | P<.001 vs. control |
| mIPSC | control | 100±16 | 9 |  |
|  | α2δ1 | 184±33 | 11 | P<.05 vs. control |
|  | α2δ3 | 376±47 | 10 | P<.001 vs. control |
|  | Cd^2+^control | 43±4 | 7 | P<.001 vs. control |
|  | Cd^2+^ α2δ3 | 44±6 | 6 | P<.001 vs. control |

Figure 4 I. Mean mEPSC frequency (normalized to control mean value with respective toxin).

| toxin | group | mean±S.E.M., % | n, neurons | K-W ANOVA,  H and P values | Dunn’s test,  p value |
| --- | --- | --- | --- | --- | --- |
| agatoxin | control | 100±9 | 10 | n.s. |  |
|  | α2δ1 | 96±19 | 11 |  |  |
|  | α2δ3 | 120±31 | 8 |  |  |
| conotoxin | control | 100±30 | 10 | H_3,22_=11.6  P<.01 |  |
|  | α2δ1 | 390±65 | 6 |  | P<.01 vs. control  P<.01 vs. α2δ3 |
|  | α2δ3 | 117±26 | 6 |  | n.s. vs. control  P<.01 vs. α2δ1 |

Figure 4 J. Mean mIPSC frequency (normalized to control mean value with respective toxin).

| toxin | group | mean±S.E.M., % | n, neurons | K-W ANOVA,  H and P values | Dunn’s test,  p value |
| --- | --- | --- | --- | --- | --- |
| agatoxin | control | 100±14 | 10 | H_3,33_=21.8  P<.001 |  |
|  | α2δ1 | 121±40 | 11 |  | n.s. vs. control  P<.001 vs. α2δ3 |
|  | α2δ3 | 751±128 | 12 |  | P<.001 vs. control  P<.001 vs. α2δ1 |
| conotoxin | control | 100±26 | 10 | n.s. |  |
|  | α2δ1 | 197±59 | 8 |  |  |
|  | α2δ3 | 199±80 | 8 |  |  |

**Figure 5-1.**

Figure 5 B. Mean live HA fluorescence intensity (normalized to mean values in sister control cultures)

| group | mean±S.E.M., % | n, neurons | Mann-Whitney U test, P value |
| --- | --- | --- | --- |
| control | 100±10 | 34 | P<.001 |
| shRNA α2δ1 | 24±2 | 36 |  |

Figure 5 C. Levels of α2δ1 expression (normalized to mean values in sister control cultures).

| group | mean±S.E.M., % | n, preparations | t-test,  t and P values |
| --- | --- | --- | --- |
| control | 100±0 | 4 | t=6.14, P<.001 |
| shRNA α2δ1 | 52±8 | 4 |  |

Figure 5 E. Mean mEPSC frequency in hippocampal neurons (normalized to the mean value in respective controls).

| group | frequency ± S.E.M. (Hz) | n, neurons | Mann-Whitney U test, P value |
| --- | --- | --- | --- |
| control | 0.65 ± 0.12 | 7 | P<.05 |
| shRNA α2δ1 | 0.29 ± 0.05 | 7 |  |
| control | 3.69 ± 1.03 | 10 | n.s. |
| GFP | 3.17 ± 0.55 | 11 |  |

Figure 5 G. Mean mEPSC amplitude in hippocampal neurons (normalized to the mean value in respective controls).

| group | mean amplitude ± S.E.M. (pA) | n, neurons | Mann-Whitney U test, P value |
| --- | --- | --- | --- |
| control | 21.4 ± 2.2 | 12 | n.s. |
| shRNA α2δ1 | 20.2 ± 2.2 | 8 |  |
| control | 17.9 ± 0.57 | 10 | n.s. |
| GFP | 23.44 ± 2.9 | 11 |  |

Figure 5 K. Mean spontaneous firing rate at DIV28 one week after lentiviral infection in hippocampal cultures grown on MEAs (control n=10 MEAs; α2δ1 overexpression n=6 MEAs; α2δ1 knock-down n=5 MEAs) (normalized for each culture to own pre-infection mean value at DIV21 (taken as 100%)).

| DIV | group | mean±S.E.M., % | n, channels | 2-way ANOVA,  F and P values | Duncan’s test,  P value |
| --- | --- | --- | --- | --- | --- |
| 21 | control | 100±9 | 366 | group factor  F_2,1329_=11.8 P<.001  time factor  F_1,1329_=2.3; P=.13  interaction  F_2,1329_=9.7; P<.001 |  |
|  | α2δ1 | 100±12 | 158 |  | P<.001 vs. α2δ1 DIV28 |
|  | shRNA α2δ1 | 100±9 | 159 |  |  |
| 28 | control | 103±11 | 342 |  |  |
|  | α2δ1 OE | 203±31 | 192 |  | P<.001 vs. control  P<.001 vs. shRNA α2δ1 |
|  | shRNA α2δ1 | 54±6 | 118 |  | P<0.05 vs. control  P<.001 vs. α2δ1 |

**Figure 6-1.**

Figure 6 B. Live fluorescence intensity of HA-tagged α2δ3 subunits in HEK293T cells (data were normalized to the mean in cells expressing α2δ3-HA with scrambled shRNA).

| group | mean±S.E.M., % | n, cells | Mann-Whitney U test, P value |
| --- | --- | --- | --- |
| α2δ3-HA + scrambled shRNA | 100±5 | 44 | P<.001 |
| α2δ3-HA + shRNA | 51±5 | 49 |  |

Figure 6 D. Live fluorescence intensity of α2δ3-HA in hippocampal neurons at DIV9 (data were normalized to the mean in neurons expressing α2δ3-HA with scrambled shRNA).

| group | mean±S.E.M., % | n, neurons | Mann-Whitney U test, P value |
| --- | --- | --- | --- |
| α2δ3-HA + scrambled shRNA | 100±21 | 9 | P<.05 |
| α2δ3-HA + shRNA | 32±7 | 10 |  |

Figure 6 F. Western blots of HEK293T cells expressing the HA-tagged α2δ3 subunit together with the scrambled shRNA or the α2δ3 shRNA (data were normalized to the mean in preparations with scrambled shRNA).

| group | mean±S.E.M., % | n, preparations | Mann-Whitney U test, P value |
| --- | --- | --- | --- |
| α2δ3-HA + scrambled shRNA | 100±12 | 10 | P<.001 |
| α2δ3-HA + shRNA | 44±3 | 10 |  |

Figure 6 I. Western blots of hippocampal cultures expressing the HA-tagged α2δ3 subunit together with the scrambled shRNA or the α2δ3 shRNA (data were normalized to the mean in preparations with scrambled shRNA).

| group | mean±S.E.M., % | n, preparations | Mann-Whitney U test, P value |
| --- | --- | --- | --- |
| α2δ3-HA + scrambled shRNA | 100±14 | 5 | P<.05 |
| α2δ3-HA + shRNA | 51±3 | 5 |  |

Figure 6 K. Mean mIPSC frequency in hippocampal neurons (normalized to the mean value in respective controls).

| group | frequency ± S.E.M. (Hz) | | n, neurons | Mann-Whitney U test, P value |
| --- | --- | --- | --- | --- |
| control | | 0.9 ± 0.18 | 11 | P<.05 |
| shRNA α2δ3 | | 0.35 ± 0.07 | 13 |  |
| control | | 1.95 ± 0.37 | 7 | n.s. |
| GFP | | 1.84 ± 0.29 | 8 |  |

Figure 6 M. Mean mIPSC amplitude in hippocampal neurons (normalized to the mean value in respective controls).

| group | Mean ± S.E.M., %  mean amplitude (pA) | n, neurons | Mann-Whitney U test, P value |
| --- | --- | --- | --- |
| control | 63.4 ± 10.1 | 11 | P<.05 |
| shRNA α2δ3 | 58,9 ± 5.7 | 13 |  |
| control | 57.1 ± 9 | 7 | n.s. |
| GFP | 74.48 ± 12 | 8 |  |

Figure 6 Q. Mean spontaneous firing rate at DIV14 one week after lentiviral infection in hippocampal cultures grown on MEAs (control n=7 MEAs; α2δ3 overexpression n=5 MEAs; α2δ3 knock-down n=4 MEAs) (normalized to the mean value in controls).

| group | mean±S.E.M., % | n, channels | one-way ANOVA,  F and P values | Duncan’s test,  P value |
| --- | --- | --- | --- | --- |
| control | 100±25 | 90 | group factor  F_2,303_=20.5 P<.001 |  |
| α2δ1 OE | 279±40 | 109 |  | P<.001 vs. control  P<.001 vs. shRNA α2δ1 |
| shRNA α2δ1 | 46±8 | 107 |  | P<0.05 vs. control  P<.001 vs. α2δ1 OE |

**Figure 7-1.**

Figure 7 D. Mean number of glutamatergic synapses per µm (normalized to the mean values in control sister cultures at respective DIV).

| DIV | group | mean±S.E.M., % | n, preparations | n, ROIs | K-W ANOVA,  H and P values | post hoc test,  P value |
| --- | --- | --- | --- | --- | --- | --- |
| 14-17 | control | 100±4 | 2 | 43 | H_2,142_=3.6 P=.17 |  |
|  | α2δ1 | 108±4 |  | 51 |  |  |
|  | α2δ3 | 111±5 |  | 48 |  |  |
| 18-24 | control | 100±5 | 4 | 104 | H_2,348_=33.8 P<.001 |  |
|  | α2δ1 | 136±4 |  | 131 |  | P<.001 vs. control  n.s. vs. α2δ3 |
|  | α2δ3 | 123±5 |  | 113 |  | P<.001 vs. control  n.s. vs. α2δ1 |

Figure 7 E. Mean number of GABAergic synapses per µm (normalized to the mean values in control sister cultures at respective DIV).

| DIV | group | mean±S.E.M., % | n,  preparations | n, ROIs | K-W ANOVA,  H and P values | post hoc test,  P value |
| --- | --- | --- | --- | --- | --- | --- |
| 14-17 | control | 100±4 | 3 | 69 | H_2,201_=15.8 P<.001 |  |
|  | α2δ1 | 114±5 |  | 67 |  | n.s. vs. control  P=.09 vs. α2δ3 |
|  | α2δ3 | 129±5 |  | 65 |  | P<.001 vs. control  P=.09 vs. α2δ1 |
| 18-24 | control | 100±5 | 3 | 68 | H_2,242_=25.6 P<.001 |  |
|  | α2δ1 | 124±8 |  | 74 |  | n.s. vs. control  P<.01 vs. α2δ3 |
|  | α2δ3 | 165±11 |  | 100 |  | P<.001 vs. control  P<.01 vs. α2δ1 |

Figure 7 G. Mean fluorescence intensity in HA-positive puncta for presynaptic proteins in transfected rat hippocampal cultures overexpressing either α2δ1, or α2δ3 subunits (data were normalized to respective mean values (taken as 100%) in sister control cultures).

| protein | group | mean±S.E.M., % | n, neurons | ANOVA,  F and P values | Bonferroni test,  P value |
| --- | --- | --- | --- | --- | --- |
| Bassoon | control | 100±2 | 138 | F_2,273_=72.8 P<.001 |  |
|  | α2δ1 | 146±3 | 62 |  | P<.001 vs. control  P<.001 vs. α2δ3 |
|  | α2δ3 | 118±3 | 76 |  | P<.001 vs. control  P<.001 vs. α2δ1 |
| RIM | control | 100±3 | 56 | F_2,109_ =20.1 P<.001 |  |
|  | α2δ1 | 127±4 | 21 |  | P<.001 vs. control |
|  | α2δ3 | 118±3 | 35 |  | P<.001 vs. control |
| VGlut1 | control | 100±3 | 96 | F_2,189_ =18.5 P<.001 |  |
|  | α2δ1 | 122±4 | 53 |  | P<.001 vs. control |
|  | α2δ3 | 131±5 | 43 |  | P<.001 vs. control |
| VGAT | control | 100±7 | 89 | F_2,175_ =9.6 P<.001 |  |
|  | α2δ1 | 83±10 | 41 |  |  |
|  | α2δ3 | 138±9 | 48 |  | P<.01 vs. control  P<.001 vs. α2δ1 |
| Ca_V_2.1 | control | 100±3 | 57 | F_2,111_ =11.8 P<.001 |  |
|  | α2δ1 | 119±4 | 25 |  | P<.001 vs. control |
|  | α2δ3 | 118±4 | 32 |  | P<.001 vs. control |
| Ca_V_2.2 | control | 100±2 | 120 | F_2,237_ =14.3 P<.001 |  |
|  | α2δ1 | 122±4 | 53 |  | P<.001 vs. control |
|  | α2δ3 | 114±3 | 67 |  | P<.01 vs. control |

**Figure 8-1**.

Figure 8 D. Mean axon length in GAD67-positive interneurons (normalized to mean values in control sister cultures).

| group | mean±S.E.M., % | n, preparations | n, neurons | K-W ANOVA,  H and P values | Dunn's test,  P value |
| --- | --- | --- | --- | --- | --- |
| control | 100±6 | 3 | 28 | H_2,84_=42.6  P<.001 |  |
| α2δ1 | 82±6 |  | 27 |  | n.s. vs. control  P<.001 vs. α2δ3 |
| α2δ3 | 179±11 |  | 29 |  | P<.001 vs. control  P<.001 vs. α2δ1 |

Figure 8 E. Mean number of axonal branches in GAD67-positive interneurons (normalized to mean values in control sister cultures).

| group | mean±S.E.M., % | n, preparations | n, neurons | K-W ANOVA,  H and P values | Dunn's test,  P value |
| --- | --- | --- | --- | --- | --- |
| control | 100±9 | 3 | 28 | H_2,84_=39.8  P<.001 |  |
| α2δ1 | 86±8 |  | 27 |  | n.s. vs. control  P<.001 vs. α2δ3 |
| α2δ3 | 219±15 |  | 29 |  | P<.001 vs. control  P<.001 vs. α2δ1 |
